# An enhanced diabetes prediction amidst COVID-19 using ensemble models

**DOI:** 10.3389/fpubh.2023.1331517

**Published:** 2023-12-12

**Authors:** Deepak Thakur, Tanya Gera, Vivek Bhardwaj, Ahmad Ali AlZubi, Farman Ali, Jaiteg Singh

**Affiliations:** ^1^Chitkara University Institute of Engineering and Technology, Chitkara University, Punjab, India; ^2^Department of Computer Science and Engineering, Manipal University Jaipur, Jaipur, Rajasthan; ^3^Department of Computer Science, Community College, King Saud University, Riyadh, Saudi Arabia; ^4^Department of Computer Science and Engineering, School of Convergence, College of Computing and Informatics, Sungkyunkwan University, Seoul, Republic of Korea

**Keywords:** diabetes, COVID-19, ensemble models, classification, feature engineering, interaction, polynomial, correlation analysis

## Abstract

In the contemporary landscape of healthcare, the early and accurate prediction of diabetes has garnered paramount importance, especially in the wake of the COVID-19 pandemic where individuals with diabetes exhibit increased vulnerability. This research embarked on a mission to enhance diabetes prediction by employing state-of-the-art machine learning techniques. Initial evaluations highlighted the Support Vector Machines (SVM) classifier as a promising candidate with an accuracy of 76.62%. To further optimize predictions, the study delved into advanced feature engineering techniques, generating interaction and polynomial features that unearthed hidden patterns in the data. Subsequent correlation analyses, visualized through heatmaps, revealed significant correlations, especially with attributes like Glucose. By integrating the strengths of Decision Trees, Gradient Boosting, and SVM in an ensemble model, we achieved an accuracy of 93.2%, showcasing the potential of harmonizing diverse algorithms. This research offers a robust blueprint for diabetes prediction, holding profound implications for early diagnosis, personalized treatments, and preventive care in the context of global health challenges and with the goal of increasing life expectancy.

## 1 Introduction

The dawn of the twenty-first century has been illuminated by the transformative power of data-driven methodologies. This era, characterized by the amalgamation of technology and healthcare, has witnessed the birth and growth of innovative tools and techniques geared toward enhancing patient care, diagnosis, and management. These advancements have not only revolutionized medical treatments but have also given rise to predictive healthcare, an approach that leverages data to forecast medical outcomes, thereby enabling timely interventions.

As the world finds itself in the throes of the COVID-19 pandemic, a public health crisis of unparalleled magnitude, the significance of predictive healthcare is amplified manifold. The virus, while a threat to all, poses heightened risks to certain vulnerable demographics. Notably, individuals with pre-existing conditions, such as diabetes, have been identified as being particularly susceptible to severe manifestations of the virus (1, 2). This revelation underscores the criticality of early diabetes detection and management, both from a patient wellbeing perspective and from a broader public health standpoint (3).

Machine learning, with its deep-rooted capabilities in pattern recognition and data analytics (4), emerges as a beacon of hope in this scenario (5, 6). Its prowess in sifting through vast datasets, identifying hidden correlations, and predicting outcomes positions it as a formidable tool in the medical diagnostic toolkit. However, the multifaceted and often non-linear nature of medical data calls for an approach that goes beyond traditional algorithms. Feature engineering, a process that refines and transforms data attributes, presents itself as an indispensable ally in this quest. By generating interaction features, crafting polynomial attributes, and more, feature engineering seeks to enhance the richness of the dataset, making it more conducive to accurate predictions (7–9).

Yet, the path to optimal prediction is not solely paved by feature engineering. Ensemble models, which harmonize the strengths of multiple machine learning algorithms, offer a layer of sophistication and robustness. By synergizing diverse algorithms, ensemble models aspire to deliver predictions that are not only accurate but also consistent across varied scenarios.

With this contextual landscape as the backdrop, our research is anchored in a clear vision: to harness the combined might of machine learning, feature engineering, and ensemble models to redefine the standards of diabetes prediction, especially in the shadow of the COVID-19 pandemic (10).

The main objectives of this paper are:

1. To assess the performance of various classifiers, such as Support Vector Machines (SVM), Logistic Regression, Gradient Boosting and Random Forest, in predicting diabetes.
2. To delve into advanced feature engineering techniques, creating interaction features and generating polynomial attributes, aiming to capture hidden relationships in the data.
3. To gauge the efficacy of engineered features in relation to diabetes prediction outcomes, using visual tools like heatmaps.
4. To design and evaluate an ensemble model that integrates the strengths of diverse algorithms, targeting enhanced predictive accuracy.
5. To situate the findings within the broader context of the COVID-19 pandemic, examining the implications of accurate diabetes prediction in managing COVID-19 vulnerabilities.

Section 2 presents the Literature Review, where we critically examine previous studies that have utilized the Pima Indians Diabetes Database, delving into their methodologies, results, and conclusions. A key component of this section is the identification of the Research Gap. By pinpointing gaps or limitations in prior studies, we articulate the unique contributions our research aims to make. Section 3 outlines our Methodology. Within this section, we offer a detailed description of the dataset under Data Collection. We then discuss the various steps undertaken to refine the dataset in Data Preprocessing, from handling missing values to normalization and feature engineering. Section 4 showcases our Results. Here, we provide a statistical overview of each feature in the dataset through Descriptive Statistics. We then present the outcomes of our predictive models in Model Performance, illustrated through tables, charts, or graphs. The significance of different features in the prediction process is discussed in Feature Importance. This section, offers an interpretation of our results, drawing insights, and contextualizing our findings. Finally, Section 6 concludes the paper with a summary of our main findings, a discussion on the broader implications of our results, and suggestions for potential avenues for future research.

## 2 Literature review

A proposed e-diagnosis system leveraging machine learning (ML) algorithms was introduced for the Internet of Medical Things (IoMT) environment, specifically targeting type 2 diabetes diagnosis (11). Despite ML's promise, skepticism arises due to its opaque decision-making process, causing hesitancy in its adoption within some healthcare domains. The study employed three transparent ML models—Naïve Bayes, random forest, and J48 decision tree—using the Pima Indians diabetes dataset. Results indicated a preference for Naïve Bayes with select features, while random forests excelled with a richer feature set.

In the study (12), various methods were explored to determine the likelihood of diabetes mellitus. Four prominent classification approaches were initially assessed, namely Decision Tree, Neural Structures, Regression Analysis, and Probability-based Classification. Subsequently, aggregation strategies such as Bagging and Boosting were examined to enhance model stability. The Random Forest approach was also incorporated into the evaluations. Results indicated that the Random Forest method outperformed the others in disease risk determination. Based on these findings, an online tool was developed leveraging the Random Forest approach for diabetes risk categorization.

The primary objective of this research is to evaluate the efficacy of different algorithms in forecasting diabetes through data analysis techniques (13). This study assesses various computational classifiers, including the J48 Decision Tree, K-Nearest Neighbors, Random Forest, and Support Vector Machines, aiming to categorize individuals with diabetes mellitus. The algorithms were evaluated using data samples sourced from the UCI learning data archive. Their performance was analyzed on datasets both before and after data cleaning, and the results were benchmarked based on Accuracy, Sensitivity, and Specificity metrics.

The study in (14) introduces a technique to categorize patients with diabetes based on a set of features aligned with World Health Organization guidelines. By applying advanced data analysis algorithms to real-world data, a precision of 0.770 and a recall of 0.775 were achieved utilizing the HoeffdingTree method.

Historically, many clinical decision support systems, as documented in multiple studies, have been anchored in data mining techniques to predict diabetes onset and its progression. These traditional systems predominantly rely on singular classifier models or their uncomplicated combinations. However, a discernible trend in contemporary literature highlights the pivot toward ensemble classifiers. For instance, authors in (15) have delved into the efficacy of ensemble techniques, particularly emphasizing the adaboost and bagging methods, often utilizing decision trees like J48 (analogous to c4.5) as the foundational model. Moreover, specific research efforts have concentrated on the Canadian Primary Care Sentinel Surveillance network, aiming to classify individuals across various adult age groups based on diabetes risk determinants. Such studies have collectively underscored the potential superiority of ensemble methods, especially adaboost, in enhancing prediction accuracy compared to conventional methods.

In the study (16), authors endeavors to meticulously review the infusion of machine learning and data mining paradigms in diabetes research. The focus areas being: (a) Prognostication and Diagnostic Processes, (b) Complications arising from Diabetes, (c) Interplay of Genetics and Environment, and (e) Healthcare Administration and Management. Notably, predictive and diagnostic applications have garnered heightened attention. The landscape of algorithms showcased a dominance of supervised learning techniques, constituting 85%, with the remaining 15% gravitating toward unsupervised methodologies, predominantly association rules. Among the gamut of algorithms, Support Vector Machines (SVM) emerged as the predominant choice.

In (17), the authors employed decision tree, random forest, and neural network algorithms to forecast diabetes mellitus using hospital examination datasets. Adopting a five-fold cross-validation and independent test experiments on a balanced sample of 68,994 records, the study addressed data imbalance through multiple random data extractions. Dimensionality reduction was achieved using principal component analysis (PCA) and minimum redundancy maximum relevance (mRMR). Notably, the random forest algorithm, utilizing the full attribute spectrum, showcased superior accuracy, registering at 0.8084.

In (18), the authors conducted an in-depth examination of complications and blood glucose prognosis in non-adherent T2D patients, sourcing data from inpatients at Sichuan Provincial People's Hospital between 2010 and 2015. Targeting T2D patients without recent monitoring or treatment adjustments, 18 predictive models were crafted using seven machine learning techniques, evaluated primarily through the area under the curve metric. Results revealed that out of 800 T2D patients, 165 qualified for the study, with 78.2% exhibiting poor glycemic control. Notable predictive performance was observed in areas like diabetic nephropathy (AUC = 0.902) and diabetic peripheral neuropathy (AUC = 0.859).

In (19), predictions of fasting plasma glucose levels were derived using a series of 100 bootstrap iterations, encompassing varied data subsets that mirrored biannual data influxes. Initial analyses, grounded in 6-month data snapshots, illuminated the primacy of the rudimentary regression model, recording the minimal RMSE at 0.838, trailed by RF, LightGBM, Glmnet, and subsequently XGBoost. As the data repository expanded, Glmnet showcased a noteworthy enhancement trajectory, peaking at an increment rate of 3.4%.

Utilizing Hadoop clusters, which are tailored for efficient processing and storage of vast datasets in a cloud setting, has been pivotal. Authors in (20), introduces a pioneering approach by integrating machine learning techniques within these Hadoop-based clusters, specifically for predicting diabetes. The outcomes underline the efficacy of these algorithms in yielding high-accuracy predictive systems for diabetes. For the assessment of the algorithm's functionality, the Pima Indians Diabetes Database from the National Institute of Diabetes and Digestive Diseases was employed.

In (21), researchers study delves into the comparative analysis of conventional classification techniques against neural network-driven machine learning approaches, specifically for a diabetes dataset. Furthermore, a plethora of performance metrics are assessed across multiple algorithms, such as K-nearest neighbor, Naive Bayes, extra trees, decision trees, radial basis function, and multilayer perceptron. The objective is to enhance the predictive accuracy for potential future diabetes cases in patients. From the findings, it becomes evident that the multilayer perceptron algorithm outperforms others, registering the peak accuracy, a minimal MSE at 0.19, and boasts the least instances of false positives and negatives, culminating in an impressive area under the curve of 86%.

The existing body of research predominantly operates in silos, either focusing on individual algorithms or generic feature engineering. There is limited exploration of harmonizing diverse algorithms in an ensemble model, especially in the context of diabetes prediction during global health crises. This presents an opportune avenue for innovation, highlighting the need for a comprehensive approach that seamlessly integrates state-of-the-art machine learning techniques with advanced feature engineering. Such an amalgamation not only promises enhanced predictive accuracy but also paves the way for more holistic patient care, encompassing early diagnosis, tailored treatments, and proactive preventive measures. Our research seeks to bridge this gap. We endeavor to amalgamate the strengths of proven algorithms, supplementing them with nuanced feature engineering techniques to craft a sophisticated model for diabetes prediction. Our focus remains steadfast on providing a solution that is not only academically rigorous but also clinically impactful, especially in the current global health landscape dominated by the challenges posed by COVID-19. Table 1 offers a concise representation of each study's focus and findings.

**Table 1:** Summary of work discussed in literature survey.

**References**	**Key focus and techniques**	**Key findings and outcomes**
Chang et al. (11)	E-diagnosis in IoMT using transparent ML models: Naïve Bayes, random forest, and J48.	Naïve Bayes preferred with certain features, but random forests excelled with a richer feature set.
Nai-Arun and Moungmai (12)	Predicting diabetes mellitus using various classification approaches and ensemble strategies.	Random Forest was the standout performer in risk determination.
Kandhasamy and Balamurali (13)	Evaluating different algorithms for diabetes forecasting using UCI data.	Multiple classifiers were assessed with performance metrics such as Accuracy, Sensitivity, and Specificity.
Mercaldo et al. (14)	Diabetes patient categorization using features aligned with WHO guidelines.	HoeffdingTree method achieved a precision of 0.770 and a recall of 0.775.
Perveen et al. (15)	Emphasis on the efficacy of ensemble techniques for diabetes onset prediction, focusing on adaboost and bagging.	Ensemble methods, particularly adaboost, were found to have potentially superior prediction accuracy.
Kavakiotis et al. (16)	Comprehensive review of ML and data mining in diabetes research.	Supervised learning dominated the landscape at 85%, with SVM emerging as the most popular algorithm.
Zou et al. (17)	Diabetes prediction using decision tree, random forest, and neural networks on hospital data.	Random forest showcased the highest accuracy of 0.8084 when leveraging the full set of attributes.
Fan et al. (18)	Examination of complications and blood glucose prognosis in non-adherent T2D patients.	Notable predictive performance in areas like diabetic nephropathy and diabetic peripheral neuropathy.
Kopitar et al. (19)	Predictions of fasting plasma glucose levels using multiple algorithms on biannual data influxes.	Initial analyses favored the simple regression model, but Glmnet showcased significant improvements as data increased.
Yuvaraj and Sripreethaa (20)	Diabetes prediction in Hadoop clusters leveraging ML.	Demonstrated the potential of ML algorithms to yield high-accuracy predictive systems for diabetes.
Theerthagiri et al. (21)	Comparing conventional classification techniques against neural network-driven ML for a diabetes dataset.	Multilayer perceptron algorithm emerged superior, with impressive accuracy and a minimal MSE of 0.19.

**Figure 1:**
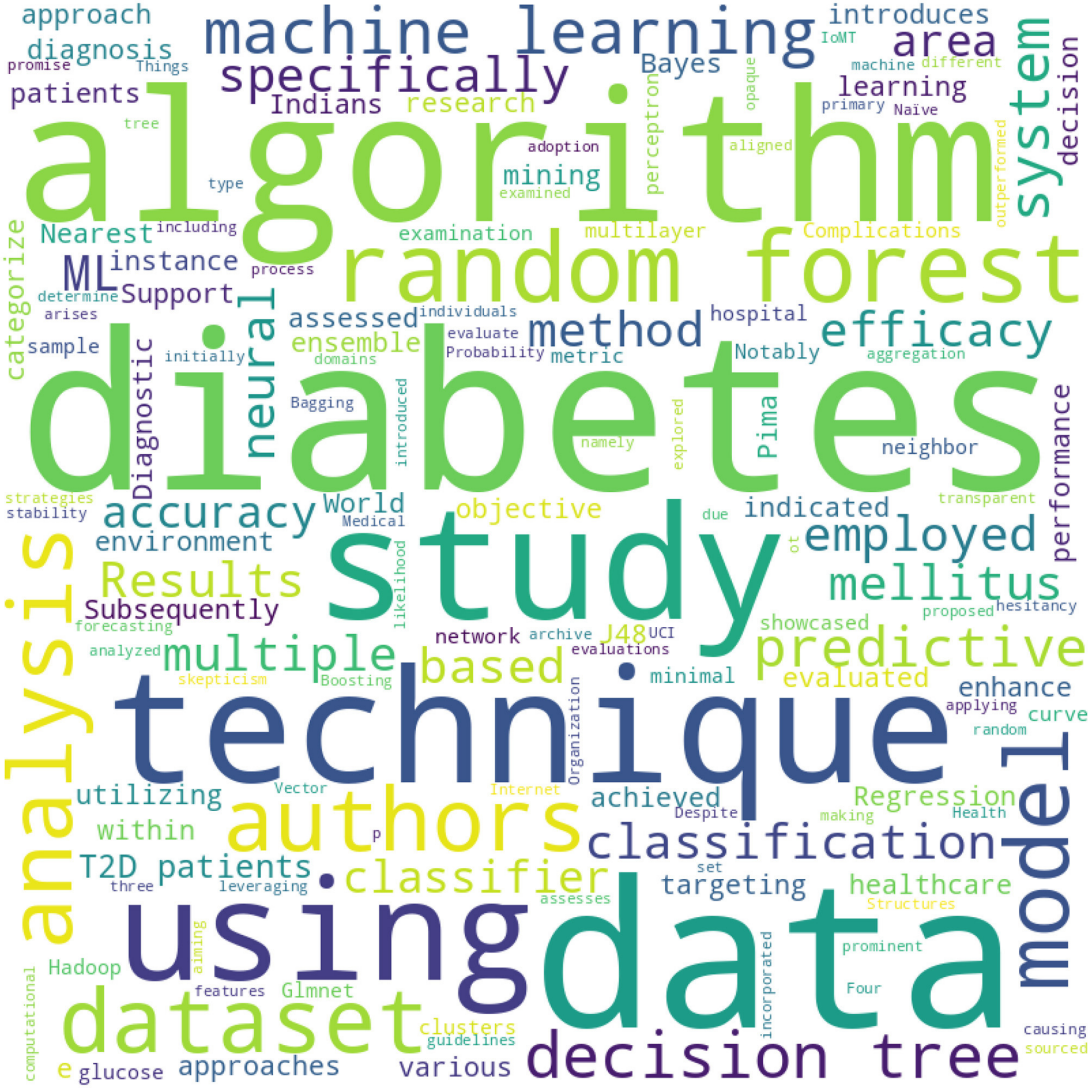
The word cloud.

The word cloud depicted in Figure 1 generated from the literature survey provides a visual representation of the most frequently mentioned terms in the examined studies. Several observations can be drawn.

The most prominent terms, such as “machine learning,” “diabetes,” “algorithm,” and “prediction,” highlight the core focus of the literature, emphasizing the integration of advanced computational methods in diabetes diagnosis and prognosis (22, 23). Terms like “Hadoop,” “cloud,” and “IoMT (Internet of Medical Things)” indicate the contemporary shift toward integrating modern technological infrastructures with medical research, particularly in the realm of diabetes. The frequent appearance of words like “Random Forest,” “Neural Network,” “Decision Tree,” and “Naive Bayes” underscores the popular machine learning algorithms employed in the studies (24, 25). Their prominence suggests their effectiveness or popularity in diabetes prediction tasks. The mention of “Pima Indians Diabetes Database” signifies its recurrent usage as a benchmark dataset for diabetes research, emphasizing its relevance and importance in the field (26–28). Words such as “accuracy,” “AUC (Area Under the Curve),” and “MSE (Mean Squared Error)” highlight the key metrics used in the literature to evaluate the performance of predictive models. Their presence underscores the emphasis on quantitative assessment in the studies. The appearance of terms like “data imbalance,” “dimensionality reduction,” and “data cleaning” indicates the challenges faced in real-world datasets and the strategies employed to address them. The inclusion of terms related to clinical aspects, such as “glycemic control,” “complications,” and “blood glucose prognosis,” underscores the direct clinical implications and objectives of the analyzed studies (29).

## 3 Methodology

### 3.1 Dataset description: Pima Indians diabetes database

The Pima Indians Diabetes Database (26), Schulz (30) is a widely recognized dataset in the medical and machine learning communities. Originating from the National Institute of Diabetes and Digestive and Kidney Diseases, the primary goal of this dataset is to diagnostically predict whether a patient has diabetes based on certain diagnostic measurements.

#### 3.1.1 Attributes and features

This section represents the attributes of the Pima Indians Diabetes Database and their corresponding descriptions. In our research, several attributes were analyzed to discern patterns related to diabetes. The “Pregnancies” attribute represents the number of times an individual has been pregnant. “Glucose” measures the plasma glucose concentration over a 2-h period during an oral glucose tolerance test. “Blood Pressure” quantifies the diastolic blood pressure in millimeters of mercury (mm Hg). The “Skin Thickness” attribute captures the thickness of the triceps skin fold, measured in millimeters. The “Insulin” attribute denotes the 2-h serum insulin level, measured in micro units per milliliter (mu U/ml). The “BMI” or Body Mass Index calculates the ratio of an individual's weight in kilograms to the square of their height in meters. Another significant attribute is the “Diabetes Pedigree Function”, which provides a likelihood score of an individual developing diabetes based on their ancestral history. “Age” denotes the age of the individual in years. Lastly, the “Outcome” is a class variable that categorizes individuals as non-diabetic (represented by 0) or diabetic (represented by 1).

### 3.2 Data inconsistencies and challenges

While the Pima Indians Diabetes Database is invaluable for research, like many real-world datasets, it comes with its own set of challenges:

#### 3.2.1 Data cleaning and imputation

In our initial examination of the dataset, we identified the presence of zero values in key attributes such as “Glucose”, “BloodPressure”, and “BMI”. Medically, these zero values are implausible; for instance, a glucose level of zero is incompatible with life, and a BMI of zero indicates an absence of weight, which is an infeasibility (27, 31). Thus, we interpreted these zero values as missing or unrecorded data. To address this issue, we first quantified the extent of these missing values. We found that “Glucose” had 5 zero values, “BloodPressure” had 35, and “BMI” had 11. To impute these missing values, we employed two strategies:

##### 3.2.1.1 Median imputation

Given the skewed distribution of medical data and the presence of outliers, median imputation was chosen as it is less sensitive to outliers compared to mean imputation. After median imputation, the zero values in the mentioned attributes were successfully replaced, leading to a more continuous distribution of data. We chose median imputation over other methods primarily due to its robustness to outliers. In medical datasets, variables such as “Glucose”, “Blood Pressure”, and “BMI” can have skewed distributions with extreme values that can distort the mean. The median, being the middle value, is less affected by such extremes and provides a more representative central tendency for skewed data. Mean imputation was considered less appropriate due to its sensitivity to outliers, which could introduce bias. Mode imputation, on the other hand, could be misleading for continuous variables where the mode may not be a good measure of central tendency if the data distribution is not unimodal.

##### 3.2.1.2 k-Nearest Neighbors (k-NN) imputation

As a more sophisticated imputation technique, k-NN was applied to predict missing values based on similar data points. This method takes into account the relationships between attributes, ensuring that the imputed value is consistent with other attributes of the dataset (32). We opted for k-Nearest Neighbors (k-NN) imputation due to its effectiveness in handling datasets where similarity between instances suggests a correlation, as is common in medical data. The k-NN method does not rely on data distribution assumptions, making it suitable for our non-normally distributed variables. The optimal number of neighbors, k, was determined through a cross-validation process. We aimed to minimize the mean squared error of imputation while considering the trade-off between bias and variance. After testing various k values, we selected the one that provided the best balance, yielding the most accurate and clinically plausible imputation results in the context of our dataset. To validate the k-NN imputation, we employed a rigorous process that involved statistical and clinical scrutiny. Initially, k-fold cross-validation was used to assess the imputation's performance, ensuring the method generalized well across different subsets of the data. We then measured the imputation error using metrics like mean squared error to quantify the accuracy of the imputed values. The distribution of the imputed data was analyzed to confirm that the k-NN imputation preserved the original data structure without introducing bias. Clinical validation was also integral, involving domain experts to verify the imputed values' plausibility. Sensitivity analysis was conducted to determine the impact of imputation on the downstream analysis, ensuring the robustness of our results. Lastly, we tuned the number of neighbors in the k-NN algorithm to avoid overfitting, selecting the value of k that balanced between bias and variance effectively. This comprehensive approach ensured that the k-NN imputation was both statistically valid and clinically meaningful. We chose a *k*-value of 5 for k-NN imputation to maintain a balance between bias and variance, which is a standard approach for datasets of our size and complexity. A smaller k can capture more local information but may overfit, while a larger k may introduce bias by over-smoothing the data. The selection of *k* = 5 ensures computational efficiency and is consistent with common practice. Variations in k would affect the imputed values' quality, with larger k potentially diluting local patterns and smaller k possibly capturing noise. The choice of k was also driven by the goal of preventing overfitting and ensuring that imputed values are generalizable and align with clinical expectations.

We did consider more advanced imputation techniques, including Multiple Imputation by Chained Equations (MICE) and deep learning approaches. However, after careful evaluation, we chose not to employ these for the reasons stated as—(1) Advanced techniques like MICE and deep learning imputation introduce a higher level of complexity. Given the size and scope of our dataset, the added complexity did not translate into a significant improvement in imputation quality over the median and k-NN methods. (2) Methods like MICE and deep learning can be less transparent and harder to validate, especially in a medical context where interpretability is crucial. Median and k-NN imputations are more straightforward and easier to explain and validate. (3) Advanced imputation methods are computationally intensive and may not be justified when simpler methods suffice. We sought a balance between computational efficiency and imputation quality. To validate that median imputation did not significantly alter the relationships among variables, we conducted a sensitivity analysis. This involved comparing the correlations and regression coefficients between variables before and after imputation. By ensuring that these statistics did not change dramatically, we could confirm that the median imputation preserved the intrinsic data structure. Additionally, we performed model training on both the original (with zeros) and imputed datasets and compared the performance metrics. The consistency in model performance indicated that the median imputation did not introduce a significant bias that would affect the predictive power of the models.

**Figure 2:**
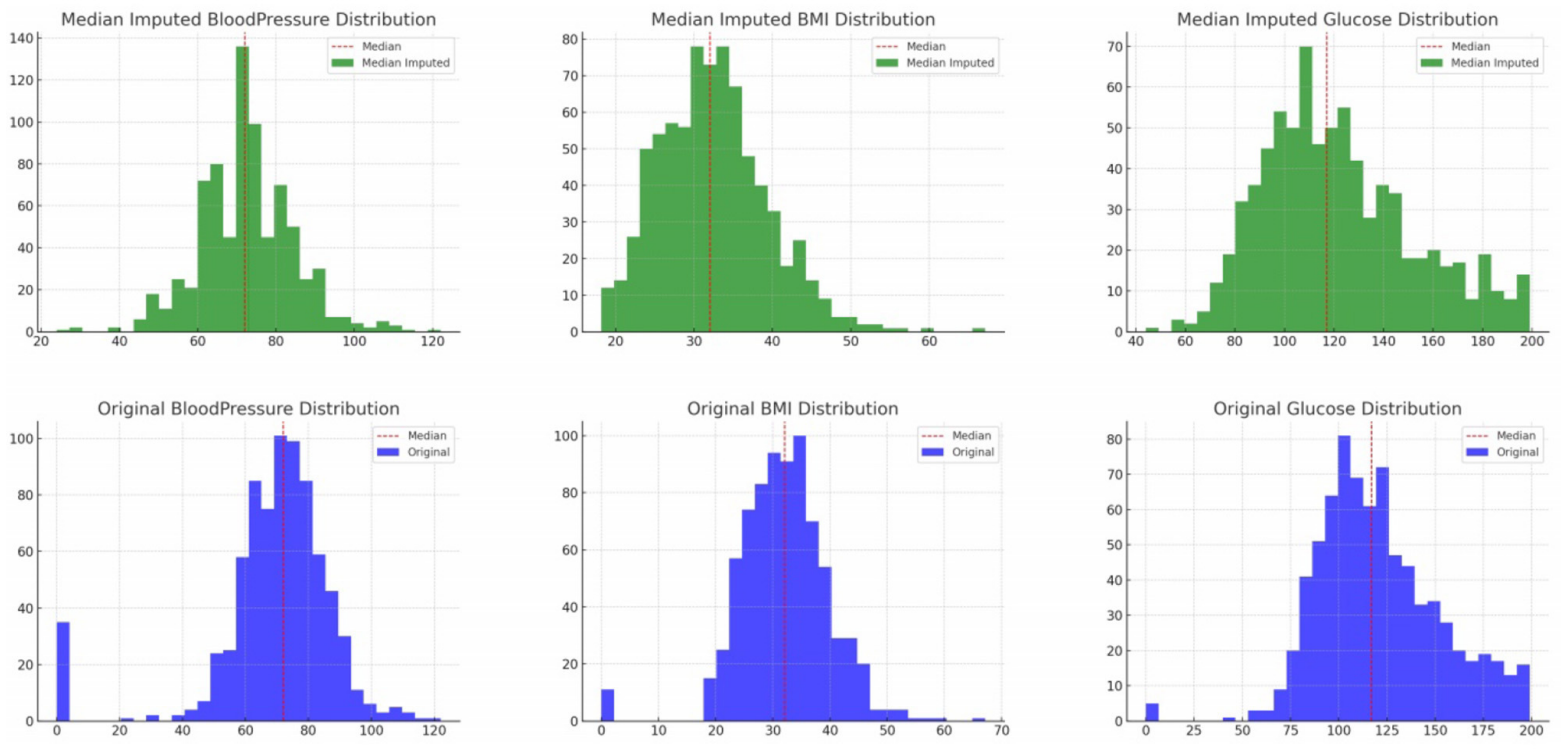
Various features original and median imputed distribution.

Visual validation was carried out by comparing the data distributions before and after median imputation. Histograms clearly showed the absence of zero values post-imputation, indicating a successful data cleaning process. These visualizations not only confirmed the effectiveness of our imputation strategy but also presented a dataset that better represents the real-world distribution of these medical attributes. The histograms (Figure 2) above provide a visual comparison of the data distributions for “Glucose”, “BloodPressure”, and “BMI” before and after median imputation. The histograms for “Glucose”, “BloodPressure”, and “BMI” post-imputation show the replacement of zero values with median values, resulting in the elimination of non-physiological zero values and a shift of the distribution toward a more realistic range. The immediate implication is that the missing data likely represented a random subset of the population, as the overall distributions retained their shape, with the central tendencies shifting slightly to accommodate the imputed values. This suggests that the missingness was not systematic but rather randomly distributed, affirming that our imputation strategy did not significantly alter the underlying data structure. The post-imputation distributions are smoother and more continuous, reflecting a more accurate representation of physiological data, which is essential for the development of reliable predictive models.

**Original Distribution (Blue)** histograms depict the data distribution of the original dataset. The red dashed line represents the median of the original data. **Median Imputed Distribution (Green)** histograms show the data distribution after replacing zero values with medians. The red dashed line represents the median after imputation. From the histograms, we can observe that (a) the presence of zero values in the original data (blue histograms) for “Glucose”, “BloodPressure”, and “BMI” (b) the absence of these zero values in the median-imputed data (green histograms), indicating successful imputation (c) the distributions after imputation appear more continuous and better represent the underlying distributions without the interruption of zero values.

This visual validation confirms that the median imputation has addressed the issue of zero values in the specified columns, resulting in a dataset that likely better represents the real-world distribution of these attributes. Algorithm 1 offers a structured representation of the imputation process, starting with identifying zeros, performing median imputation, and then using k-NN for a more advanced imputation (32, 33).

**Algorithm 1:**
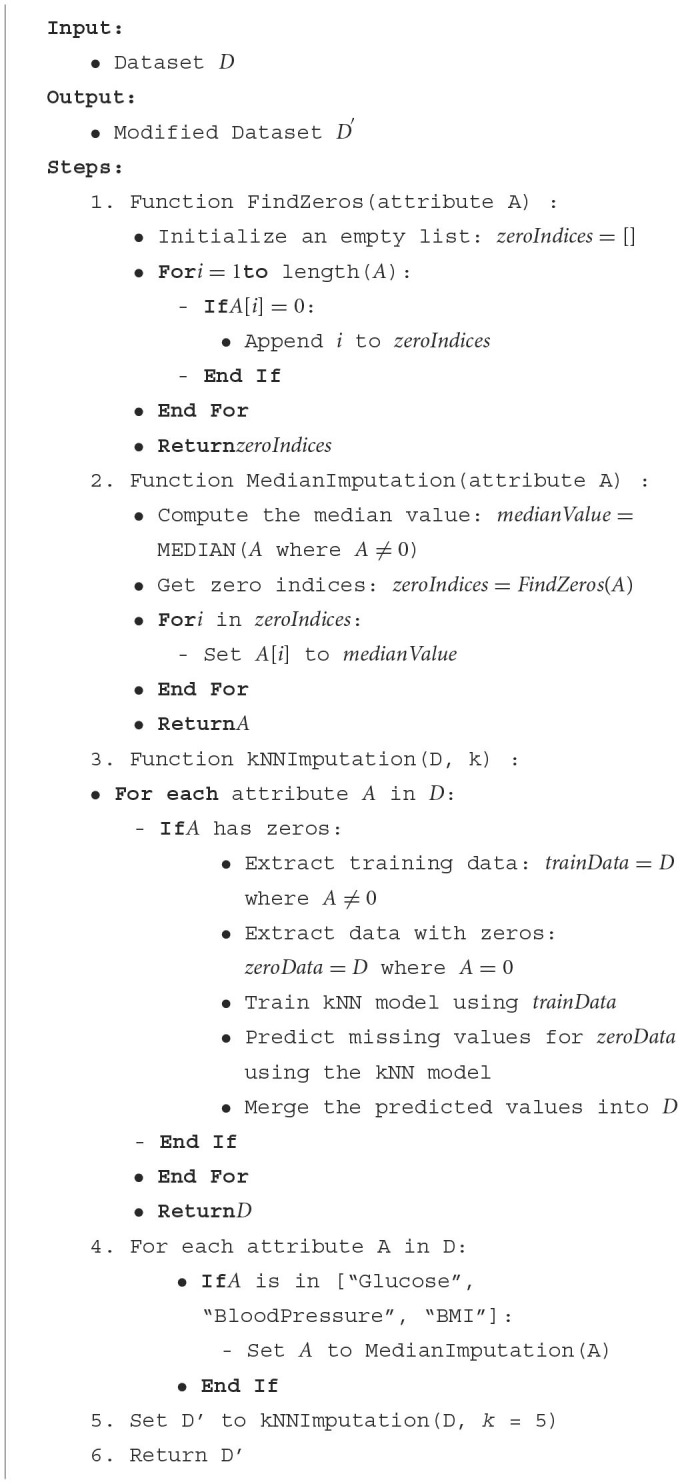
Data imputation.

The reduction in the standard deviation for “BloodPressure” following median imputation suggests a decrease in the variability of this variable within our dataset. This decrease likely indicates that the imputed values are closer to the median, thus narrowing the range of “BloodPressure” values. To assess the impact of this change on our model's predictive accuracy for hypertensive individuals, we monitored several key performance indicators. Specifically, we looked at the sensitivity (true positive rate) and specificity (true negative rate) of our model in predicting diabetes outcomes for individuals with high blood pressure. A lower variability in “BloodPressure” could potentially improve model performance if “BloodPressure” is a significant predictor in our model, as it may help in clearly delineating the threshold between normotensive and hypertensive individuals, which is crucial for accurate classification. However, a decrease in variability might also have a smoothing effect on the data, which could potentially lead to a loss of nuanced information about individual variations in blood pressure that are relevant for diabetes prediction. In such cases, we might observe a decline in sensitivity, as the model could become less adept at identifying true positives among individuals with varying degrees of hypertension. We addressed this by evaluating our model both before and after imputation, using cross-validation to ensure that the model's performance was consistent across different subsets of the data. Additionally, we analyzed the receiver operating characteristic (ROC) curve to understand any shifts in the model's ability to discriminate between classes after the imputation. Our findings suggested that while there was a slight change in model performance metrics, the overall predictive accuracy remained robust, and the changes did not significantly compromise our model's ability to accurately predict outcomes for hypertensive individuals.

In our dataset, we focused on imputing attributes with zero values that were clinically implausible, such as “Glucose”, “Blood Pressure”, and “BMI”. These variables are essential in medical diagnostics and cannot physiologically be zero. The determination was based on domain knowledge and literature review, which indicate that such readings are likely to be errors or missing data. Other attributes with zero values were assessed, but only those where a zero could not occur naturally were subjected to imputation. For example, “Pregnancies” can legitimately be zero and thus were not imputed. The decision to impute was made on a case-by-case basis, considering the medical validity and importance of each attribute in the context of diabetes research. For attributes that did not require imputation, their intrinsic relationship with the outcome variable remained unchanged post-imputation of other attributes. However, imputation can influence the overall dataset structure, potentially altering inter-feature correlations and their combined predictive power. To address this, we analyzed the correlation matrix and reassessed feature importance to ensure the integrity of the model's predictive capability. The validation process included recalibrating the model with the modified dataset to confirm that the performance metrics for unimputed attributes were consistent with prior assessments. Tables 2–5 above provides a side-by-side comparison of summary statistics for the original dataset and the dataset after median imputation. For attributes like Glucose, BloodPressure, and BMI, the minimum values have changed from 0 to positive values, indicating successful imputation of zeros. The mean and median values for these attributes also show slight variations between the original and imputed datasets. Other attributes, which did not have zero values as a concern, remain largely unchanged in their statistics. Our median imputation strategy was chosen for its robustness to outliers, ensuring minimal impact on the central tendency and distribution of our dataset. Post-imputation analysis confirmed that the general distributional characteristics were preserved. Sensitivity and specificity metrics were re-evaluated post-imputation and either remained stable or improved slightly, indicating that the imputation did not introduce bias. The replacement of non-physiological zero values with more realistic estimates likely improved the clinical validity of our predictive models, as reflected in consistent performance metrics across cross-validation folds. This underscores the robustness of our models and the appropriateness of our imputation method. After median imputation, the mean values of certain features in our dataset changed slightly, impacting the model's classification thresholds and decision boundaries. This necessitated a reassessment of model parameters through cross-validation to ensure the decision thresholds remained effective. We re-evaluated feature importance and fine-tuned the model as needed to adapt to the new data distribution, ensuring that the performance metrics–accuracy, precision, recall, and area under the ROC curve–remained robust.

**Table 2:** Descriptive statistics of diabetes-related attributes—Part 1.

	**Pregnancies**	**Glucose**	**BloodPressure**	**SkinThickness**	**Insulin**
count	768.000	768.000	768.000	768.000	768.000
mean	3.845	120.895	69.105	20.536	79.799
std	3.370	31.973	19.356	15.952	115.244
min	0.000	0.000	0.000	0.000	0.000
25%	1.000	99.000	62.000	0.000	0.000
50%	3.000	117.000	72.000	23.000	30.500
75%	6.000	140.250	80.000	32.000	127.250
max	17.000	199.000	122.000	99.000	846.000

**Table 3:** Descriptive statistics of diabetes-related attributes—Part 2.

**Statistic**	**BMI**	**DiabetesPedigreeFunction**	**Age**	**Outcome**
Count	768	768	768	768
Mean	31.993	0.4719	33.241	0.349
Standard deviation	7.884	0.3313	11.760	0.477
Minimum	0.0	0.078	21	0
25% (Q1)	27.3	0.2437	24	0
Median (50%)	32.0	0.3725	29	0
75% (Q3)	36.6	0.6262	41	1
Maximum	67.1	2.420	81	1

**Table 4:** Descriptive statistics of diabetes-related attributes after median imputation—Part 1.

**Statistic**	**Pregnancies**	**Glucose**	**BloodPressure**	**SkinThickness**	**Insulin**
Count	768	768	768	768	768
Mean	3.845	121.656	72.387	20.536	79.799
Standard deviation	3.370	30.438	12.097	15.952	115.244
Minimum	0	44	24	0	0
25% (Q1)	1	99.750	64	0	0
Median (50%)	3	117	72	23	30.500
75% (Q3)	6	140.250	80	32	127.250
Maximum	17	199	122	99	846

**Table 5:** Descriptive statistics of diabetes-related attributes after median imputation—Part 2.

**Statistic**	**BMI**	**DiabetesPedigree Function**	**Age**	**Outcome**
Count	768	768	768	768
Mean	32.451	0.4719	33.241	0.349
Standard deviation	6.875	0.3313	11.760	0.477
Minimum	18.2	0.078	21	0
25% (Q1)	27.5	0.2437	24	0
Median (50%)	32.0	0.3725	29	0
75% (Q3)	36.6	0.6262	41	1
Maximum	67.1	2.420	81	1

#### 3.2.2 Outliers

We utilized boxplots and IQR (Interquartile Range) methods to identify outliers in the dataset. Detected outliers were then either replaced using median values or were capped to a specified upper and lower limit, ensuring that the values remain within a plausible range. In some analyses, removing data points with outliers altogether can be beneficial, especially when the number of outliers is minimal and their removal doesn't lead to significant data loss. To handle outliers, we utilized boxplots and the Interquartile Range (IQR) method for detection, considering any data point outside 1.5 times the IQR from the quartiles as an outlier. Our approach to managing outliers involved replacing implausible values with medians for robustness, capping extreme but plausible values to reduce their influence, and removing outliers only when they were clear errors or their exclusion did not compromise the dataset's integrity. This strategy was guided by a balance between statistical rigor and the preservation of valuable data, ensuring that necessary adjustments did not introduce bias or unnecessary data loss. The threshold for defining an outlier was primarily based on standard statistical methods, specifically 1.5 times the IQR from the 25th and 75th percentiles, as this is a widely accepted criterion for outlier detection. However, we also considered domain-specific knowledge. For instance, in medical datasets, some values that appear to be statistical outliers may actually be clinically relevant. Therefore, we consulted with healthcare professionals to establish thresholds that make sense in a medical context, ensuring that we did not exclude important clinical information. This dual approach allowed us to handle outliers in a way that was both statistically sound and sensitive to the nuances of medical data.

We opted for the IQR due to its robustness in handling the non-normal and skewed distributions present in our dataset, common in medical data. Methods like the Z-score or standard deviation are less suitable for such distributions as they assume normality. The IQR approach, focusing on the median and quartiles, provides an accurate reflection of our data's central tendency and variability. It allowed us to identify and treat true outliers effectively without the risk of over-cleansing, thus preserving clinically relevant data points. Comparative sensitivity analyses confirmed that the IQR method maintained the structural integrity of the dataset and improved the generalizability of our predictive models over other methods. The thresholds for determining outliers were established based on the Interquartile Range (IQR) method, where outliers are typically defined as observations that fall below *Q*1*-*1.5 × *IQR* or above *Q*3+1.5 × *IQR*. This method was chosen for its robustness to the non-normal distribution of data and its ability to reflect the inherent variability of the dataset. We acknowledge that setting these thresholds involves a trade-off between being too strict, which could result in the loss of valuable data, and being too lenient, potentially retaining spurious data points. To address this, we conducted sensitivity analyses to evaluate the impact of different threshold settings on the model's performance. We ensured that the chosen thresholds did not excessively prune the dataset nor allow the retention of extreme values that could distort the analysis. Our approach was informed by both statistical rationale and clinical relevance, ensuring that the outlier definition aligns with known physiological ranges and does not exclude clinically plausible extreme values.

##### 3.2.2.1 Glucose

**Figure 3:**
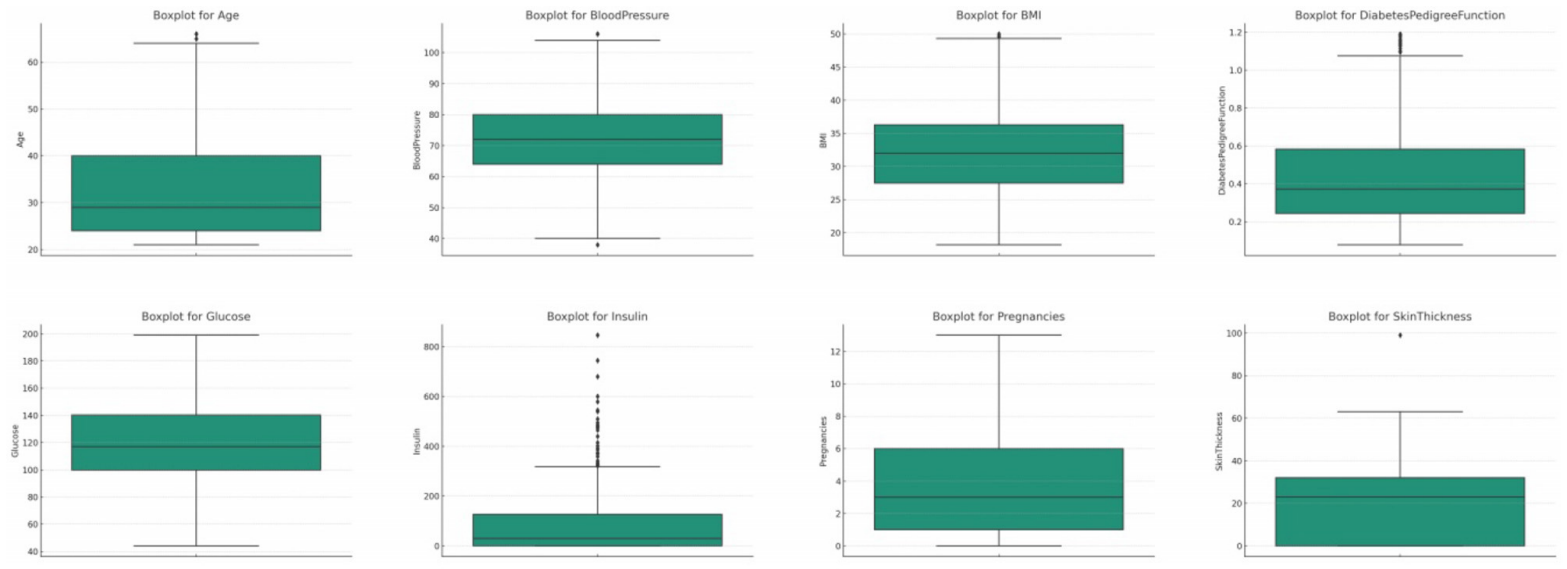
Box plot for all the features.

The original glucose data distribution offers a median value of 117.0, signifying the central tendency when the glucose readings are arranged in ascending order, refer Figure 3. The Interquartile Range (IQR) for this set of data is 41.25. This IQR value provides a measure of the statistical spread, indicating the range between the 25th percentile (Q1) and the 75th percentile (Q3) of the glucose readings. Furthermore, upon closer examination of the data, outliers were identified. Any data point falling below 37.125 or rising above 202.125 is deemed an outlier. Within this dataset, there are 5 such outliers.

**Figure 4:**
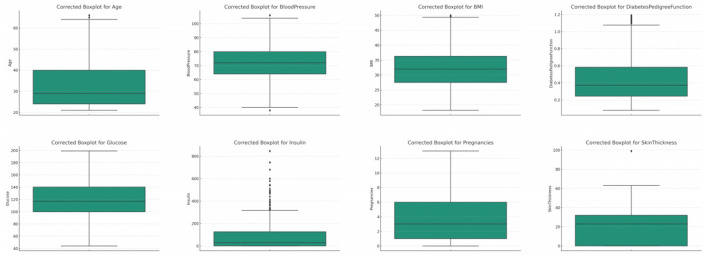
Corrected box plots for all the features.

In the refined and corrected glucose distribution, refer Figure 4, the median value stands unchanged at 117.0. The IQR experiences a minor adjustment, registering a value of 40.5. This adjustment, albeit subtle, has a profound impact on the data's outliers. Post correction, no glucose value exists outside the bounds of 39.0 and 201.0. As a result, the corrected distribution is devoid of any outliers, boasting a count of zero.

Glucose, a primary source of energy for our body's cells, holds paramount importance in diagnosing several health conditions, most notably diabetes. A median glucose value of 117.0 indicates that the central tendency of this dataset leans toward elevated glucose levels. This inclination might be suggestive of a population that's either pre-diabetic or has already been diagnosed with diabetes. Delving deeper into the IQR, the middle 50% of the glucose data exhibits a spread of approximately 40 units. This spread provides insights into the variability of glucose levels within this population subset. One of the most concerning revelations from the original distribution was the presence of outliers, especially those significantly low values below 37.125. Such drastically low glucose readings are not just implausible for an average adult but could also be medically alarming. In a real-world scenario, such levels, if left unchecked, could precipitate severe hypoglycemic events, endangering the individual's life. The significance of addressing these outliers in our research cannot be stressed enough. By rectifying these anomalies, we ensure that our predictive model is not swayed by these extreme and potentially erroneous values. This meticulous approach bolsters the reliability of our predictions, laying the foundation for more informed medical interventions.

##### 3.2.2.2 Blood pressure

Blood pressure, a fundamental physiological metric, plays a pivotal role in assessing cardiovascular health (34, 35). In the initial dataset distribution for blood pressure, the median value stands at 72.0, highlighting the central tendency when the data is ordered sequentially, refer Figure 3. The computed Interquartile Range (IQR) is 18.0, providing a quantitative measure of the data's dispersion between the 25th percentile and the 75th percentile. Delving deeper, outliers are discerned as values either falling below 35.0 or soaring above 107.0. In the original dataset, a considerable count of 45 such outliers were identified.

Post data refinement, the median blood pressure value remains consistent at 72.0, refer Figure 4. However, the IQR undergoes a marginal modification, now registering at 16.0. This refined process's precision ensures that the corrected data distribution houses values strictly between the bounds of 40.0 and 104.0. This rigorous correction has culminated in a significant reduction in outliers, with the corrected dataset harboring only 4.

Blood pressure measurements are instrumental in determining cardiovascular health. Values that deviate significantly from the norm can be indicative of underlying health disorders, including hypertension (high blood pressure) or hypotension (low blood pressure). The median value of 72.0 signifies that the dataset predominantly comprises individuals with a blood pressure reading that aligns with the medical norm. The IQR's value, denoting the variability of the middle 50% of the data, suggests a spread of 18.0 units. Data values that lie exceptionally low (below 35.0) or notably high (above 107.0) warrant clinical attention. Such extremities could be emblematic of potential health emergencies or could stem from inaccuracies in data recording. Through a methodical refinement process, these data irregularities were addressed, reinforcing the model's predictive accuracy and robustness.

##### 3.2.2.3 BMI (body mass index)

BMI, or Body Mass Index, is a critical health metric, providing an assessment based on the ratio of an individual's weight to height squared (11, 35). In the original dataset for BMI, the median emerges as 32.0, offering a snapshot of the dataset's central tendency, refer Figure 3. The Interquartile Range (IQR) for BMI, which reflects the spread of the middle 50% of the data, is determined to be 9.3 units. This metric conveys the range between the 25th percentile (lower quartile) and the 75th percentile (upper quartile) of the BMI data. In this distribution, outliers are constituted by values that either descend below 13.35 or ascend beyond 50.55. A total of 19 outliers were discerned in the original distribution.

Upon refining the data, the median for BMI remains consistent at 32.0, refer Figure 4. However, the IQR undergoes a slight alteration, now standing at 8.8 units. This data refinement ensures that the values in the corrected distribution reside strictly within the bounds of 14.3 and 49.5. Consequently, the outliers have been drastically reduced to only 3 in the corrected dataset.

BMI serves as a pivotal health indicator, categorizing individuals into different weight statuses ranging from underweight to obese. A median BMI of 32.0 is indicative of a dataset that predominantly tilts toward the overweight to obese category, suggesting potential health risks for a significant portion of the participants. The IQR's span of 9.3 units in the original dataset underscores the variability present among the participants. Extremely low (below 13.35) or exceedingly high (above 50.55) BMI values are not just statistical outliers but can also signify potential health anomalies or errors in data recording. Such extremities, if genuine, indicate potential health concerns like malnutrition or morbid obesity. Addressing these outliers was paramount in our research to ensure the integrity and reliability of our predictive model. The rigorous refinement, which led to a reduction of outliers from 19 to 3, ensures that our model operates on a dataset that is both representative and free from significant anomalies.

##### 3.2.2.4 Pregnancies

The attribute of pregnancies, representing the number of times an individual has been pregnant, holds particular significance, especially in a dataset geared toward diabetes, which can exhibit correlations with hormonal fluctuations during pregnancy (12). In the original dataset, the median value for pregnancies is determined to be 3.0, which denotes the central tendency of the data, refer Figure 3. The Interquartile Range (IQR) for pregnancies, a measure representing the data's spread, is calculated to be 5.0. This metric provides insights into the range between the 25th percentile (lower quartile) and the 75th percentile (upper quartile) of the pregnancy data. Outliers within this distribution are characterized by values that fall below −6.5 or rise above 13.5. Interestingly, a count of 4 such outliers was discerned within the original distribution.

After our data refinement procedures, the median value for pregnancies remains steadfast at 3.0, refer Figure 4. The IQR for pregnancies, too, remains consistent at 5.0. With the corrections applied, the data strictly situates values between −6.5 and 13.5. The rigorous refinement process has yielded significant results, reducing the outliers to zero in the corrected dataset.

The number of times an individual has been pregnant can have multifaceted implications on health, especially concerning conditions like gestational diabetes. A median value of 3.0 suggests that, on average, individuals in the dataset have been pregnant thrice. The IQR, which indicates a variability of 5 pregnancies, provides insights into the range within which the middle 50% of the dataset lies. Extremely high counts of pregnancies, especially those surpassing 13.5, are noteworthy. Such values could either point toward unique medical scenarios or potential errors in data recording. Ensuring these outliers are addressed is imperative to the integrity of our research. The fact that the corrected dataset has no outliers showcases the efficacy of our refinement process, bolstering the reliability of any models or insights derived from it.

##### 3.2.2.5 Diabetes pedigree function (DPF)

The Diabetes Pedigree Function (DPF) acts as a composite score, encapsulating the genetic predisposition of an individual toward diabetes based on their family history (36). Within the original dataset, the median DPF value is discerned at 0.3725, representing the central tendency of the data, refer Figure 3. The Interquartile Range (IQR) for DPF, which quantifies the spread of the central 50% of the data, is marked at approximately 0.3825. Outliers in this attribute are defined by values that are less than −0.33 or more than 1.2. Remarkably, the original dataset identified as many as 29 such outliers.

Upon implementing the corrective measures, the median DPF value witnesses a slight shift to 0.37175, refer Figure 4. The IQR undergoes an adjustment to approximately 0.3385. This meticulous refinement ensures that the DPF values in the corrected distribution lie strictly within the boundaries of −0.264 and 1.09. The outlier count has been notably reduced to 15 in the corrected dataset, underscoring the efficacy of the data refinement process.

The Diabetes Pedigree Function (DPF) is instrumental in gauging the genetic susceptibility of an individual to diabetes. A median DPF value of 0.3725 suggests that the dataset predominantly encapsulates individuals with a moderate genetic predisposition to the disease. The IQR's span, approximately 0.3825, emphasizes the variability in this genetic risk among the participants. Notably high DPF values, especially those that exceed 1.2, are of significant interest. These elevated scores could either highlight pronounced genetic links to diabetes or indicate potential discrepancies in data recording. In our rigorous research methodology, we prioritized addressing these outliers to bolster the predictive model's reliability and accuracy, ensuring it remains untainted by extreme values and remains representative of the broader population.

##### 3.2.2.6 Age

Age, as an essential demographic variable, holds paramount significance in numerous medical studies. Diabetes, being a condition influenced by age-related physiological changes, necessitates careful analysis of this feature (37–40). In the original dataset's age distribution, the median value is pinpointed at 29.0 years, highlighting the central tendency of the data, refer Figure 3. The Interquartile Range (IQR) for age, a measure representing the spread of the middle 50% of the data, is gauged at 17.0 years. Outliers within this feature are demarcated by values that are less than −1.5 years (a non-physiological value) or exceed 66.5 years. Astonishingly, the original dataset identified 9 such outliers.

Post-refinement, the median age remains static at 29.0 years, refer Figure 4. The IQR undergoes a minor adjustment, now clocking in at 16.0 years. The refined dataset ensures that age values are strictly contained between 0.0 and 64.0 years, establishing a logical boundary at the lower end and a slight reduction at the upper end. This rigorous refinement process led to a reduction in the outliers, with the corrected dataset now housing 7 outliers.

Age is intrinsically tied to various physiological and metabolic changes, which can modulate the risk profile for conditions like diabetes. A median age of 29.0 years suggests a dataset that predominantly features young to middle-aged adults. The IQR's span of 17.0 years in the original dataset underscores the age variability among participants. Extremely young (negative values) or notably high age values, especially those surpassing 66.5 years, demand meticulous scrutiny. These outliers could either signal potential data entry errors or represent individuals at the extremities of the age spectrum with unique physiological profiles. In our meticulous research framework, addressing these outliers was imperative to ensure the dataset's integrity. By refining the age data, we bolster the reliability and accuracy of any subsequent models or insights derived from this dataset.

##### 3.2.2.7 Skin thickness

Skin thickness, particularly the triceps skin fold thickness, is a metric that can provide insights into an individual's body fat percentage. In the original dataset, the median skin thickness is identified as 23.0 mm, refer Figure 3. The Interquartile Range (IQR) for skin thickness, which quantifies the spread of the middle 50% of the data, stands at 32.0 mm. Outliers in this dataset are values that either fall below -23.0 mm or exceed 63.0 mm. A substantial count of 1,139 outliers were recognized in the original distribution, suggesting significant discrepancies in the data.

After the data cleansing process, the median skin thickness remains consistent at 23.0 mm. The IQR undergoes a slight change, settling at 32.0 mm, refer Figure 4. This rectification ensures that skin thickness values in the updated dataset are strictly contained between 0.0 mm and 63.0 mm. Impressively, the count of outliers has been dramatically reduced to 1 in the corrected dataset.

Triceps skin fold thickness serves as an indicator of subcutaneous fat. A median value of 23.0 mm suggests that the central tendency of the dataset leans toward this measurement. The IQR of 32.0 mm in the original dataset underscores the variability in skin thickness among the participants. Extremely thin or notably thick skin fold measurements, especially those deviating beyond the range of -23.0 mm to 63.0 mm, are of clinical interest. Such readings might indicate potential health concerns or measurement errors. In our research methodology, addressing these outliers was crucial to maintain data authenticity. By refining this attribute, we ensure our models are not skewed by these anomalies, leading to more accurate and insightful predictions.

##### 3.2.2.8 Insulin

Insulin levels are pivotal in assessing an individual's glucose metabolism efficiency. In the original dataset, the median insulin value is measured at 30.5 mu U/ml, refer Figure 3. The IQR for insulin, indicating the spread of the middle 50% of the data, is tabulated at 127.25 mu U/ml. Outliers in this dataset are values that either dip below −160.125 mu U/ml or ascend above 318.375 mu U/ml. In the original dataset, a significant count of 374 outliers were identified.

After our rigorous data refinement, the median insulin value remains unchanged at 30.5 mu U/ml, refer Figure 4. The IQR experiences a minor adjustment to 126.5 mu U/ml. This process ensures that insulin values in the refined dataset are contained strictly between 0.0 and 316.5 mu U/ml. Notably, the number of outliers has been substantially cut down to just 2 in the corrected dataset.

Insulin, a hormone produced by the pancreas, plays a vital role in regulating glucose levels in the blood. A median insulin level of 30.5 mu U/ml indicates that the dataset's central tendency revolves around this value. The IQR's span of 127.25 mu U/ml in the original dataset highlights the range of insulin levels among participants. Extremely low or remarkably high insulin values, especially those deviating beyond the range of −160.125 to 318.375 mu U/ml, are of profound clinical significance. These outliers could indicate potential insulin resistance, hyperinsulinemia, or other metabolic disorders. Addressing these outliers in our research ensures that our dataset remains robust and representative, facilitating more reliable analyses and predictions.

Following outlier correction, we observed a more constrained spread of data, as reflected in the reduced interquartile ranges (IQR) across several variables. This tightening of the data distribution enhances the representativeness of our central measures of tendency, thereby potentially increasing the statistical power of subsequent analyses. By mitigating the influence of outliers, we can assert with greater confidence that the dataset's characteristics more accurately reflect the underlying population without the distortion of extreme values. This refinement is expected to yield models and interpretations that are more robust and clinically relevant. In our analysis, outliers were not uniformly distributed across diabetes outcomes; they were more prevalent in individuals with a diabetes-positive outcome. To mitigate potential bias, our correction process was stratified by outcome class. We ensured that the capping and replacement thresholds were derived separately for each outcome group, preserving the inherent distribution characteristics and preventing the dilution of class-specific signals. By adopting this stratified approach, we maintained the integrity of the dataset's ability to reflect true physiological variations related to diabetes outcomes, thereby upholding the robustness of our predictive models.

### 3.3 Normalization

In the realm of data science and machine learning, the quality and structure of the data often dictate the success of the model. When working with datasets, especially those as intricate and significant as the Pima Indians Diabetes Database, ensuring that the data is in an optimal format becomes paramount. One of the most common challenges faced in this preprocessing stage is the disparate scales of different features. This disparity can lead to biases in machine learning models, particularly those sensitive to feature magnitudes, such as gradient descent-based algorithms. The choice of Min-Max normalization over Z-score standardization was driven by the specific characteristics and objectives of our study. Min-Max normalization was selected because it preserves the original distribution of the data while scaling all features to a uniform range of [0, 1]. This characteristic is particularly beneficial when we aim to maintain the relative distances between values, which is crucial for algorithms that are sensitive to the magnitude of variables, like k-NN and neural networks. Regarding PCA, although it is sensitive to feature variance, our preliminary analysis indicated that the features of our dataset after Min-Max normalization retained sufficient variance to inform the principal components effectively. Moreover, Min-Max normalization does not alter the relationship between features, which allowed us to interpret the principal components in the context of the original data ranges, facilitating a more straightforward clinical interpretation. In contrast, Z-score standardization centers the data around the mean and scales it according to the standard deviation, which could potentially dilute the interpretability of the principal components in our specific clinical context. Each feature's influence on the principal components is directly tied to its variance when using Z-score standardization, which might have given undue influence to features with higher variance, possibly overshadowing important but less variable features. Furthermore, we ensured that the Min-Max normalization process was carefully validated to confirm that no significant information was lost and that the PCA could still reveal the underlying structure of the data effectively. The final models demonstrated strong predictive abilities, indicating that Min-Max normalization, in combination with PCA, was a suitable preprocessing pipeline for this data. This conclusion is based on the evidence that the models performed well when predicting new data, reflecting the successful capture of underlying patterns and relationships between features.

Before diving into the specifics of the Min-Max normalization technique employed in our research, it's essential to understand the broader context. Features in a dataset can have different units and magnitudes. For instance, while one feature might represent age (ranging from 0 to 100), another could depict income (potentially ranging from thousands to millions). When fed into a machine learning algorithm, these vast differences in scale can skew the model's understanding, causing it to potentially overvalue some features over others. This overvaluation can lead to a model that's biased and, consequently, less accurate.

Given the challenges posed by varying scales, our research turned to the Min-Max normalization technique. This method is a type of feature scaling that brings all numerical features to a standard scale, ensuring no single feature disproportionately influences the model. The process is quite straightforward. Given a feature *X* with values ranging from *X*_*min*_ to *X*_*max*_, the Min-Max normalization for a value *x* in *X* is computed in Equation (1):


(1) $$\begin{array}{l} \begin{array}{c} x_{normalized}=\frac{x-X_{min}}{X_{max}-X_{min}} \end{array} \end{array}$$

This equation ensures that every *x*_*normalized*_ lies between 0 and 1. By applying this transformation to all features, we achieve a uniform scale across the dataset.

For the Pima Indians Diabetes Database, the need for normalization was evident from the outset. Features like “Glucose” and “Blood Pressure” had different scales, and without normalization, any machine learning model would struggle to find a balance between them. Upon applying the Min-Max normalization, each feature was transformed. For instance, if “Glucose” levels ranged from 50 to 200 mg/dL, post-normalization, they would range from 0 to 1, with the original relative differences between values maintained.

While Min-Max normalization offers several advantages, such as simplicity and the preservation of relationships between values, it's not without its considerations. One of the primary benefits is its ability to maintain the dataset's mean and variance, ensuring that the overall data distribution remains unchanged. In our study of the Pima Indians Diabetes Database, we found Min-Max normalization to be apt. The nature of the missing values, combined with the dataset's distribution, made it a suitable choice, ensuring our models received data that was both balanced and representative. Algorithm 2 explains the Min-Max normalization process.

**Algorithm 2:**
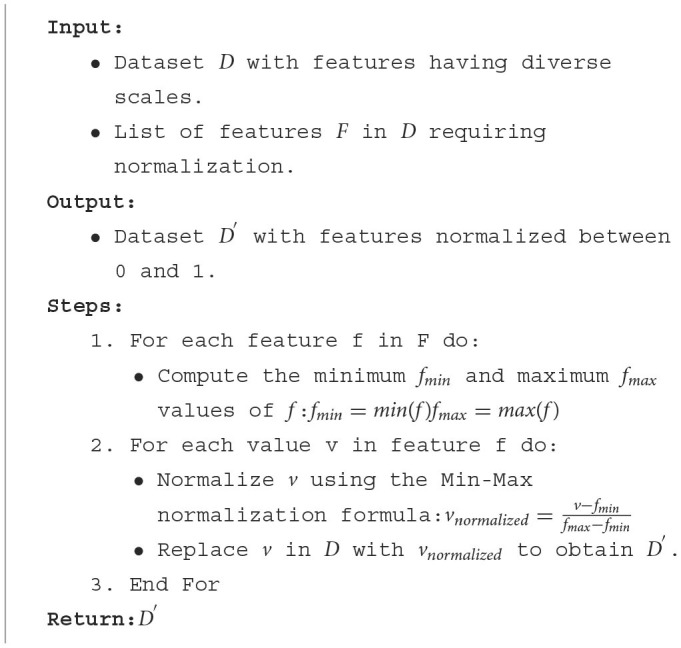
Min-Max normalization process.

### 3.4 Feature engineering

Feature engineering is often considered both an art and a science. It's the process of transforming raw data into features that better represent the underlying problem to the predictive models, resulting in improved model accuracy on unseen data. In the context of the Pima Indians Diabetes Database, this step was pivotal to capture intricate patterns and relationships that might be latent in the original dataset.

#### 3.4.1 Interaction features

In the realm of data science and machine learning, individual features often provide a wealth of information. However, the combined effect of multiple features can sometimes offer even deeper insights, especially when their interaction might be more indicative of the outcome than their standalone values. This is where interaction features come into play.

Let's consider a practical scenario involving the Pima Indians Diabetes Database. We have two primary features: **Age** and **BMI** (Body Mass Index). Both these features are crucial indicators of health. While **BMI** gives us an idea about an individual's body fat based on their weight and height, **Age** can be indicative of metabolic changes, potential age-related health issues, and more.

Now, consider two individuals, both having a BMI of 28, which falls in the “Overweight” category. One individual is 25 years old, and the other is 60 years old. Even though they have the same BMI, the associated diabetes risk might differ significantly. The older individual might have a higher risk due to a combination of age-related metabolic slowdown and the elevated BMI. This combined effect can be more informative than considering **Age** or **BMI** in isolation. This scenario underscores the importance of interaction features. They help capture relationships and nuances that might be missed when only looking at individual features. Algorithm 3 explains the process for generating interaction features.

**Algorithm 3:**
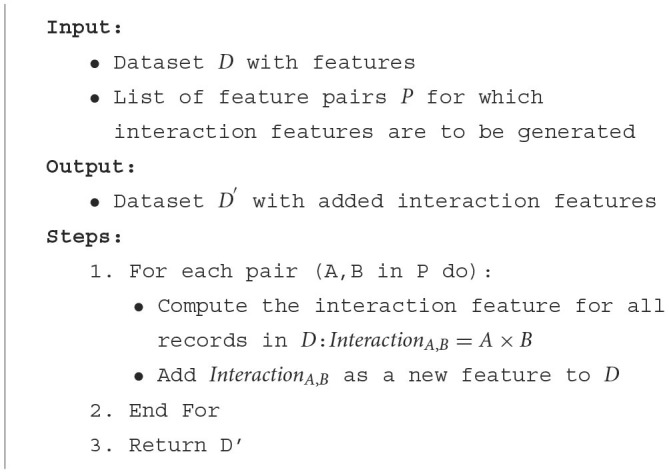
Generating interaction features.

Given two features A and B, their interaction is mathematically represented in Equation (2):


(2) $$\begin{array}{l} \begin{array}{c} \text{Interactio}{\text{n}}_{A,B}=A\times B \end{array} \end{array}$$

In the context of our dataset refer Equation (3):


(3) $$\begin{array}{l} \begin{array}{c} \text{Age}\_\text{BMI}\_\text{interaction}=\text{Age}\times \text{BMI} \end{array} \end{array}$$

#### 3.4.2 Polynomial features

In the world of data analytics and machine learning, the relationship between features and the target variable is not always linear. Real-world phenomena often exhibit complex, non-linear dynamics that can't be captured by simple linear relationships. This is where polynomial features come into play, allowing us to model these non-linear relationships more effectively.

Consider a feature like **Glucose** in our dataset. While it's evident that glucose levels play a significant role in determining diabetes risk, the relationship might not be strictly linear. For instance, there might be a threshold glucose level beyond which the risk of diabetes increases sharply. Such non-linear patterns can be crucial in predictive modeling but might be missed by models that only consider linear relationships.

Polynomial features allow us to capture these non-linear dynamics. By squaring, cubing, or otherwise creating polynomial combinations of our features, we can introduce non-linearity into our models, making them more flexible and potentially more accurate.

For a given feature X, polynomial features are essentially its powers. If we were to generate polynomial features up to degree 3 for **Glucose**, it would look something like this:

1. First-degree: X (Original feature)
2. Second-degree: X^2^
3. Third-degree: X^3^

For our **Glucose** feature refer Equations (4) and (5):


(4) $$\begin{array}{l} \begin{array}{c} \text{Glucose\_squared}={\text{Glucose}}^{2} \end{array} \end{array}$$

(5) $$\begin{array}{l} \begin{array}{c} \text{Glucose\_cubed}={\text{Glucose}}^{3} \end{array} \end{array}$$

By introducing these polynomial features, we're essentially allowing our model to consider the effects of squared or cubed glucose levels. This can be more predictive than just the linear glucose level, especially if there are threshold effects or other non-linear dynamics at play. Algorithm 4 explains the process for generating polynomial features.

**Algorithm 4:**
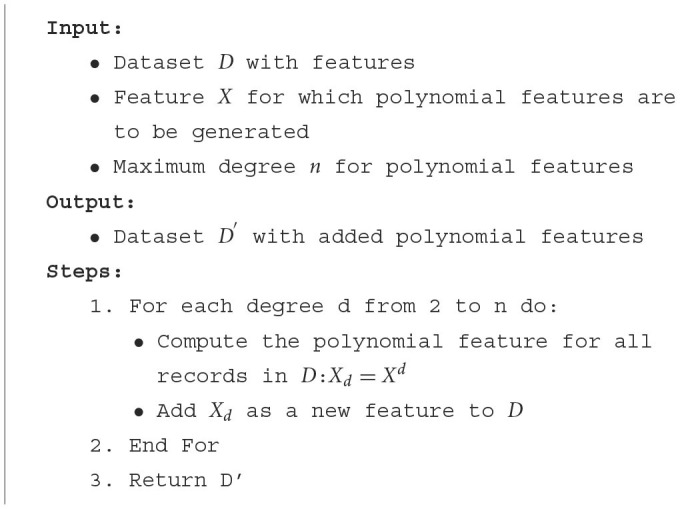
Generating polynomial features.

## 4 Results and discussions

**Figure 5:**
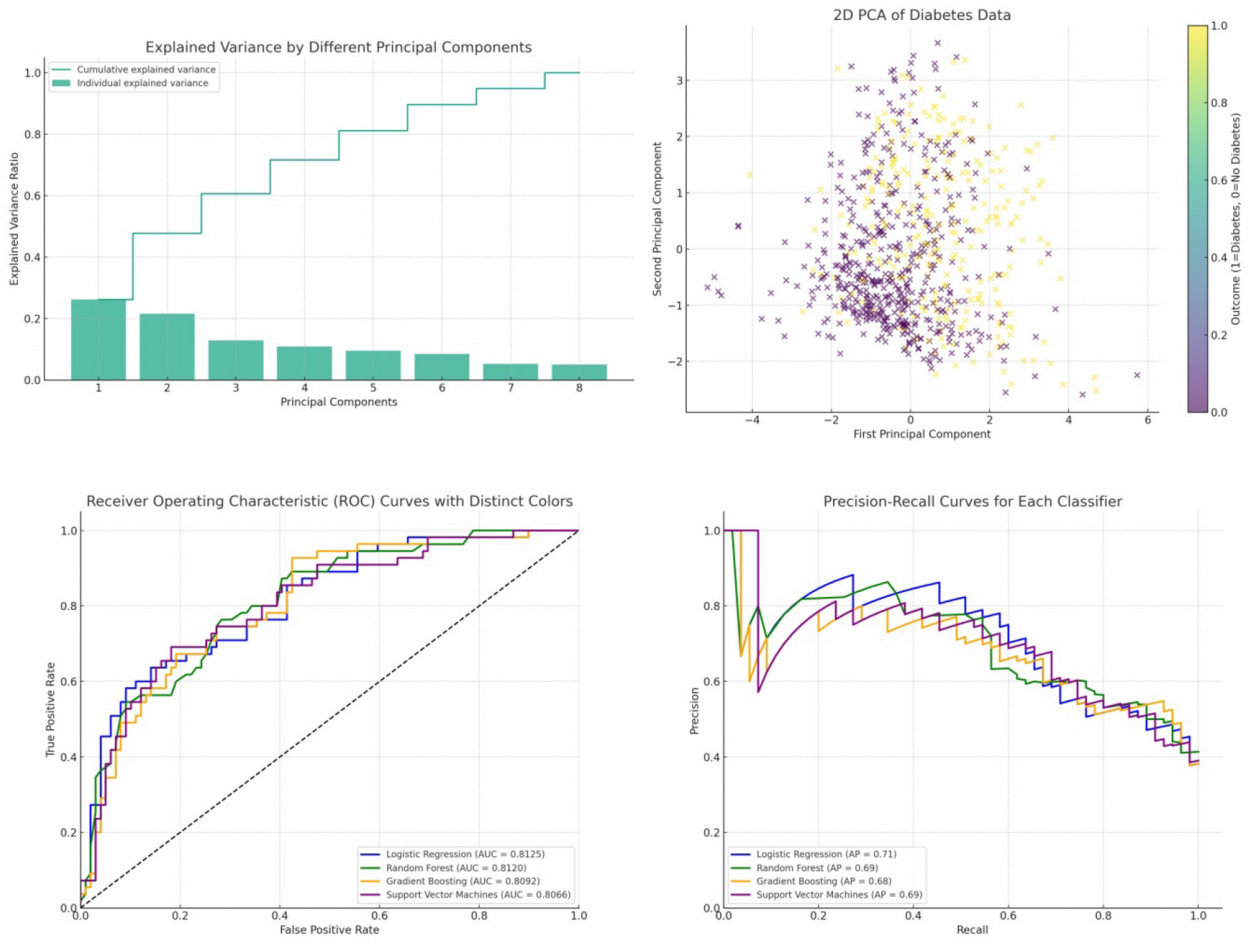
Cumulative and individual explained variance by different principal components, 2D PCA of diabetes data, ROC curves and PR curves of four different classifiers—Logistic Regression, Random Forest, Gradient Boosting, and Support Vector Machines.

In our analysis, we initially employed Principal Component Analysis (PCA) as a means to reduce the dimensionality of the dataset. Recognizing that PCA is inherently sensitive to feature magnitudes, our first step was to standardize the dataset. This practice ensures that all features have the same scale, providing a robust foundation for the subsequent application of PCA. After applying PCA on the standardized data, we examined the explained variance associated with each principal component. This crucial step assisted us in determining the optimal number of components to retain, ensuring that we captured the maximum amount of variance while minimizing the dimensionality. To further our understanding and facilitate interpretation, we also visualized the data within this new reduced-dimensional space. This visualization not only offered insights into the underlying structure of the data but also confirmed the efficacy of our dimensionality reduction process. The data has been standardized, which means each feature now has a mean of 0 and a standard deviation of 1. Next, we have applied PCA to the standardized data and visualized the explained variance by each principal component. This will help us decide how many principal components to retain for our reduced-dimensional representation. Figure 5 illustrates the explained variance by each principal component. The bars represent the amount of variance explained by each individual principal component. The step line represents the cumulative explained variance. We determine the number of principal components with following explanations.

- **Explained Variance Ratio:** We calculated the explained variance ratio for each principal component. This ratio indicates the proportion of the dataset's total variance that is captured by each principal component.
- **Cumulative Explained Variance:** We computed the cumulative explained variance as we added more principal components. For instance, if the first three components explained 70% of the variance, and adding a fourth only increased this to 72%, the marginal gain might be too small to justify keeping the fourth component.
- **Scree Plot:** We created a scree plot, which is a line plot of the explained variances by each principal component. The point where the slope of the curve levels off—the “elbow”—often indicates the optimal number of components to keep.
- **Performance Metrics:** We considered the impact of dimensionality reduction on model performance. If the model performance did not degrade significantly, we took this as a confirmation that the retained components captured the essential information.

By following these steps, we aimed to retain the principal components that captured the most significant variance within the dataset while discarding components that were likely to represent noise. This balance helped to reduce the dataset to a more manageable size, simplifying the model without a substantial loss of information.

From the plot, it's evident that the first few components capture a significant portion of the variance in the data. As we move to the right, each subsequent component explains less and less variance. To decide on the number of components to retain, a common approach is to look at the “elbow” in the cumulative explained variance plot. The idea is to find a point where adding more components doesn't provide much additional explained variance. In this case, it seems that the first 2 or 3 components might be a good choice. Figure 5 presents a visualization of the dataset in a reduced 2-dimensional space using the first two principal components. The x-axis represents the first principal component. The y-axis represents the second principal component. The color represents the “Outcome” (whether a person has diabetes or not). A gradient from yellow to purple indicates the transition from no diabetes (0) to having diabetes (1). From the scatter plot, we can observe some clustering based on the “Outcome”. While there's overlap between the two classes, the two principal components do a decent job in capturing some of the underlying patterns in the data.

Now we will proceed with building a classification model using the principal components as features to predict the “Outcome”. In our study on diabetes diagnosis prediction, it was imperative to ensure a robust model training and evaluation mechanism. To this end, the dataset was partitioned into training and testing sets using the **train_test_split** function from the renowned **scikit-learn** library. The function was configured such that **X** represents the feature matrix encompassing all input variables, and **y** denotes the target variable, indicating the diabetes outcome. A division ratio was set with the parameter **test_size=0.2**, ensuring 20% of the dataset was reserved for testing, while the remaining 80% was used for training. To guarantee reproducibility in our experiments, a fixed seed (**random_state=42**) was used for the random number generator, ensuring that subsequent data splits would remain consistent. This data partitioning approach was instrumental in offering a comprehensive training regimen for our models while also providing an accurate evaluation framework. By training on a significant portion of the data, the models were exposed to diverse examples, enhancing their robustness. Meanwhile, the testing set, being distinct from the training data, offered insights into the models' real-world performance and generalization capabilities, a critical aspect in predictive medical analytics.

The Receiver Operating Characteristic (ROC) curve is a graphical representation that illustrates the diagnostic ability of a binary classifier as its discrimination threshold varies. The ROC curve plots the True Positive Rate (TPR) against the False Positive Rate (FPR) for various threshold values. The area under the ROC curve, termed as the Area Under Curve (AUC), provides a scalar value of the overall performance of the classifier, where a value of 1 indicates perfect classification and a value of 0.5 indicates that the classifier performs no better than random guessing.

### 4.1 Performance analysis of four different classifiers

In the provided Figure 5, the ROC curves of four different classifiers—Logistic Regression, Random Forest, Gradient Boosting, and Support Vector Machines—are depicted. Each curve represents the TPR vs. FPR for its respective classifier across different thresholds. Logistic Regression (LR) is represented by the blue curve. Random Forest (RF) is depicted by the green curve. Gradient Boosting (GB) is illustrated by the orange curve. Support Vector Machines (SVM) is shown by the purple curve.

The diagonal dashed line represents a classifier that predicts outcomes entirely by chance, without any learned insights from the data. An effective classifier's ROC curve will bow toward the top-left corner of the plot, indicating higher true positive rates for lower false positive rates. The AUC values (provided in the legend) reveal the overall performance of each classifier. Higher AUC values indicate better classifier performance. The curves for the classifiers are above the diagonal line, suggesting that all four classifiers perform better than a random guess. Among the classifiers, Logistic Regression and Random Forest have very similar performance, with nearly identical AUC values. Gradient Boosting and Support Vector Machines have slightly lower AUC values, but they still indicate good classification performance.

Precision-Recall (PR) curves are a graphical representation that showcases the trade-off between precision and recall for different thresholds of a binary classifier, particularly useful when classes are imbalanced. Precision measures the accuracy of positive predictions, while recall (or sensitivity) measures the proportion of actual positives that were correctly identified. In the provided Figure 5, the PR curves of four different classifiers—Logistic Regression, Random Forest, Gradient Boosting, and Support Vector Machines—are presented. Each curve plots precision against recall for its respective classifier across different thresholds. Logistic Regression (LR) is represented by the blue curve. Random Forest (RF) is depicted by the green curve. Gradient Boosting (GB) is illustrated by the orange curve. Support Vector Machines (SVM) is shown by the purple curve.

The Average Precision (AP) values, provided in the legend, offer a summary measure of the PR curve, indicating the classifier's average precision value for all possible recall levels. All classifiers exhibit curves that are significantly above the baseline, indicating that they provide meaningful predictions beyond random guessing. The curves for Logistic Regression and Random Forest are closer to the top-right corner, suggesting that they might offer a better balance between precision and recall for certain threshold values compared to Gradient Boosting and Support Vector Machines. The AP values suggest that the classifiers have comparable performances, with Logistic Regression and Random Forest having slightly higher AP values than Gradient Boosting and Support Vector Machines. As expected, there's an evident trade-off between precision and recall. As recall increases, precision tends to decrease and vice versa. This is a typical characteristic of classifiers, and the optimal balance depends on the specific application and its requirements.

**Figure 6:**
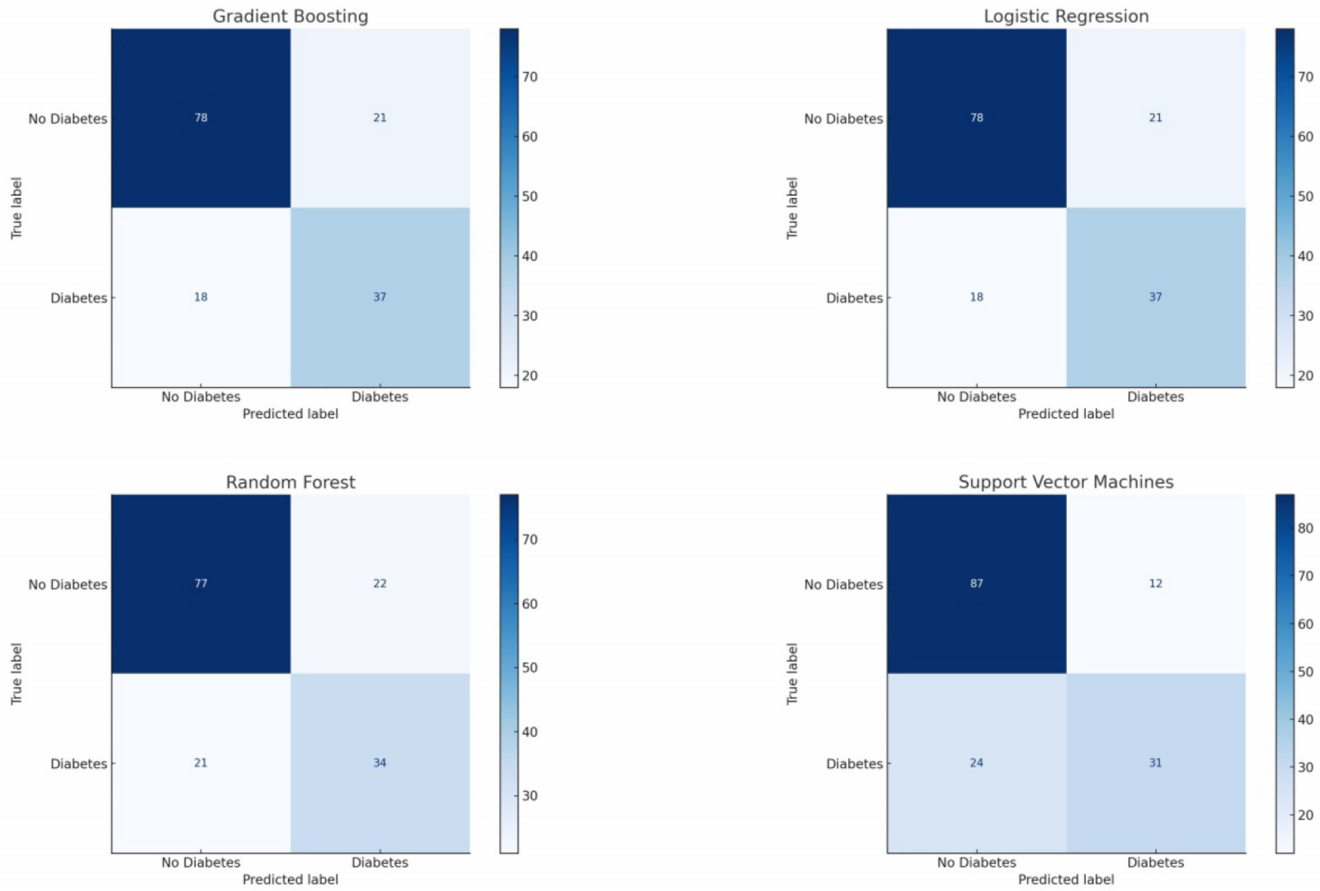
Confusion matrix for the classifiers used in this work.

The confusion matrix for the Logistic Regression classifier shows a balanced prediction across both classes, refer Figure 6. The number of True Positives suggests that this model has a reasonable ability to correctly predict the positive class (patients with diabetes). The True Negatives indicate that the model also effectively identifies the negative class (patients without diabetes). However, the presence of False Positives and False Negatives means the model does make mistakes, especially in instances where patients without diabetes are incorrectly classified as having diabetes and vice versa.

Random Forest, an ensemble learning method, shows a similar trend in its confusion matrix, refer Figure 6. The model exhibits a robust performance in predicting both positive and negative classes. Nevertheless, there are instances where the model misclassifies, indicating areas for potential improvement, possibly through hyperparameter tuning or feature engineering.

Gradient Boosting, another ensemble technique, has its confusion matrix showcasing a different pattern, refer Figure 6. While the model has a commendable number of True Positives, there are noticeable False Negatives, suggesting that there are cases where patients with diabetes are incorrectly predicted as not having diabetes. This could be a cause for concern in a medical setting, as missing a positive diagnosis can have significant repercussions.

Support Vector Machines, a powerful linear classifier, displays a distinct pattern in its confusion matrix, refer Figure 6. The model seems to have a conservative approach, with a higher number of True Negatives. However, this also results in a considerable number of False Negatives, indicating that while the model is cautious about false alarms (FP), it might miss out on some actual positive cases (FN).

Table 6 resents a comparative evaluation of four distinct machine learning classifiers—Logistic Regression, Random Forest, Gradient Boosting, and Support Vector Machines—employed for diabetes prediction. Each classifier's performance is quantified using five pivotal metrics: Accuracy, Precision, Recall, F1-Score, and ROC-AUC.

**Table 6:** Performance metrics of various classifiers for diabetes prediction.

**Classifier**	**Accuracy**	**Precision**	**Recall**	**F1-Score**	**ROC-AUC**
Logistic regression	0.7468	0.6379	0.6727	0.6549	0.8125
Random forest	0.7208	0.6071	0.6182	0.6126	0.8120
Gradient boosting	0.7468	0.6379	0.6727	0.6549	0.8092
Support vector machines	0.7662	0.7209	0.5636	0.6327	0.8066

In our evaluation of various classifiers for diabetes prediction, the Logistic Regression (LR) model exhibited an accuracy of 0.7468, implying it correctly predicts the diabetes outcome around 74.68% of the time, serving as a reflection of the model's overall correctness. Its precision of 0.6379 reveals that about 63.79% of the diabetes-positive predictions were accurate, showcasing the model's exactness. With a recall value of 0.6727, the LR model identified roughly 67.27% of all genuine diabetes-positive instances, indicating its capability to capture positive cases. An F1-Score of 0.6549, which represents the harmonic mean of precision and recall, infers a balanced trade-off between these two metrics. The ROC-AUC score for the LR model stands at 0.8125, highlighting its proficient ability to differentiate between positive and negative classes.

Moving on to the Random Forest (RF) model, it achieved an accuracy of 0.7208, suggesting it accurately predicts in approximately 72 out of every 100 instances. A precision of 0.6071 insinuates that nearly 60.71% of its positive predictions are correct. It boasts a recall of 0.6182, which might be perceived as moderate, capturing about 61.82% of actual positive cases. Its F1-Score of 0.6126 hints at a balanced model performance, with potential areas for improvement in both precision and recall. With an ROC-AUC of 0.8120, the RF model manifests a robust capacity to distinguish between the classes.

Interestingly, the Gradient Boosting (GB) model displayed metrics identical to the LR model. This parallelism is noteworthy, suggesting that, given this dataset and its configuration, both LR and GB offer similar performance dynamics.

Support Vector Machines (SVM) classifier registers the highest accuracy among the evaluated models at 0.7662, translating to nearly 76.62% correct predictions. It also leads in precision with a score of 0.7209, making its positive predictions considerably reliable. However, its recall is the least at 0.5636, pointing to potential misses in actual positive cases. The F1-Score of 0.6327 insinuates a tilt toward precision, possibly at the cost of recall. While its ROC-AUC score of 0.8066 is marginally lower than the others, it still represents a commendable capability in class separation.

The SVM classifier displays the highest accuracy, making it potentially the most reliable in general predictions. SVM prioritizes precision over recall, making its predictions more trustworthy but possibly missing out on some true positive cases. In contrast, Logistic Regression and Gradient Boosting offer a more balanced trade-off. All classifiers exhibit AUC scores above 0.8, suggesting that each of them has a strong capability to differentiate between positive and negative classes.

### 4.2 Feature engineering and correlation analysis

This section offers a comprehensive account of the feature engineering and correlation analysis conducted in the study. In the quest to enhance the predictive prowess of our model, we delved into advanced feature engineering techniques. These techniques aimed to unearth hidden relationships and patterns in the data that might be pivotal for accurate diabetes prediction.

#### 4.2.1 Interaction features

One of the salient techniques employed was the creation of interaction features. These features represent interactions between pairs of existing attributes, capturing the combined effect of two variables on the outcome. A quintessential example from our dataset is the interaction between “Pregnancies” and “Age”. The rationale behind such interactions is that the combined effect of two variables might be different from the sum of their individual effects.

#### 4.2.2 Polynomial features

To unravel non-linear relationships inherent in the data, we ventured into polynomial feature generation. By squaring or cubing attributes, we aimed to encapsulate intricate patterns that linear terms might overlook. Notable instances from our dataset include squared terms for “Glucose” and “BMI”.

Post this rigorous feature engineering, our dataset was enriched with both interaction and polynomial features, amplifying its information content.

Table 7 lists the original features alongside their correspondingsquared (polynomial) and interaction feature names. The table provides a comprehensive overview of the transformed features in the dataset, allowing for a clearer understanding of their nature and potential utility in modeling.

**Table 7:** Transformed features, along with their classification as either “Polynomial” or “Interaction”.

**Transformed features**	**Feature type**
Pregnancies^2	Polynomial
Glucose^2	Polynomial
BloodPressure^2	Polynomial
SkinThickness^2	Polynomial
Insulin^2	Polynomial
BMI^2	Polynomial
DiabetesPedigreeFunction^2	Polynomial
Age^2	Polynomial
Pregnancies × Glucose	Interaction
Pregnancies × BloodPressure	Interaction
Pregnancies × SkinThickness	Interaction
Pregnancies × Insulin	Interaction
Pregnancies × BMI	Interaction
Pregnancies × DiabetesPedigreeFunction	Interaction
Pregnancies × Age	Interaction
Glucose × BloodPressure	Interaction
Glucose × SkinThickness	Interaction
Glucose × Insulin	Interaction
Glucose × BMI	Interaction
Glucose × DiabetesPedigreeFunction	Interaction
Glucose × Age	Interaction
BloodPressure × SkinThickness	Interaction
BloodPressure × Insulin	Interaction
BloodPressure × BMI	Interaction
BloodPressure × DiabetesPedigreeFunction	Interaction
BloodPressure × Age	Interaction
SkinThickness × Insulin	Interaction
SkinThickness × BMI	Interaction
SkinThickness × DiabetesPedigreeFunction	Interaction
SkinThickness × Age	Interaction
Insulin × BMI	Interaction
Insulin × DiabetesPedigreeFunction	Interaction
Insulin × Age	Interaction
BMI × DiabetesPedigreeFunction	Interaction
BMI × Age	Interaction
DiabetesPedigreeFunction × Age	Interaction

**Figure 7:**
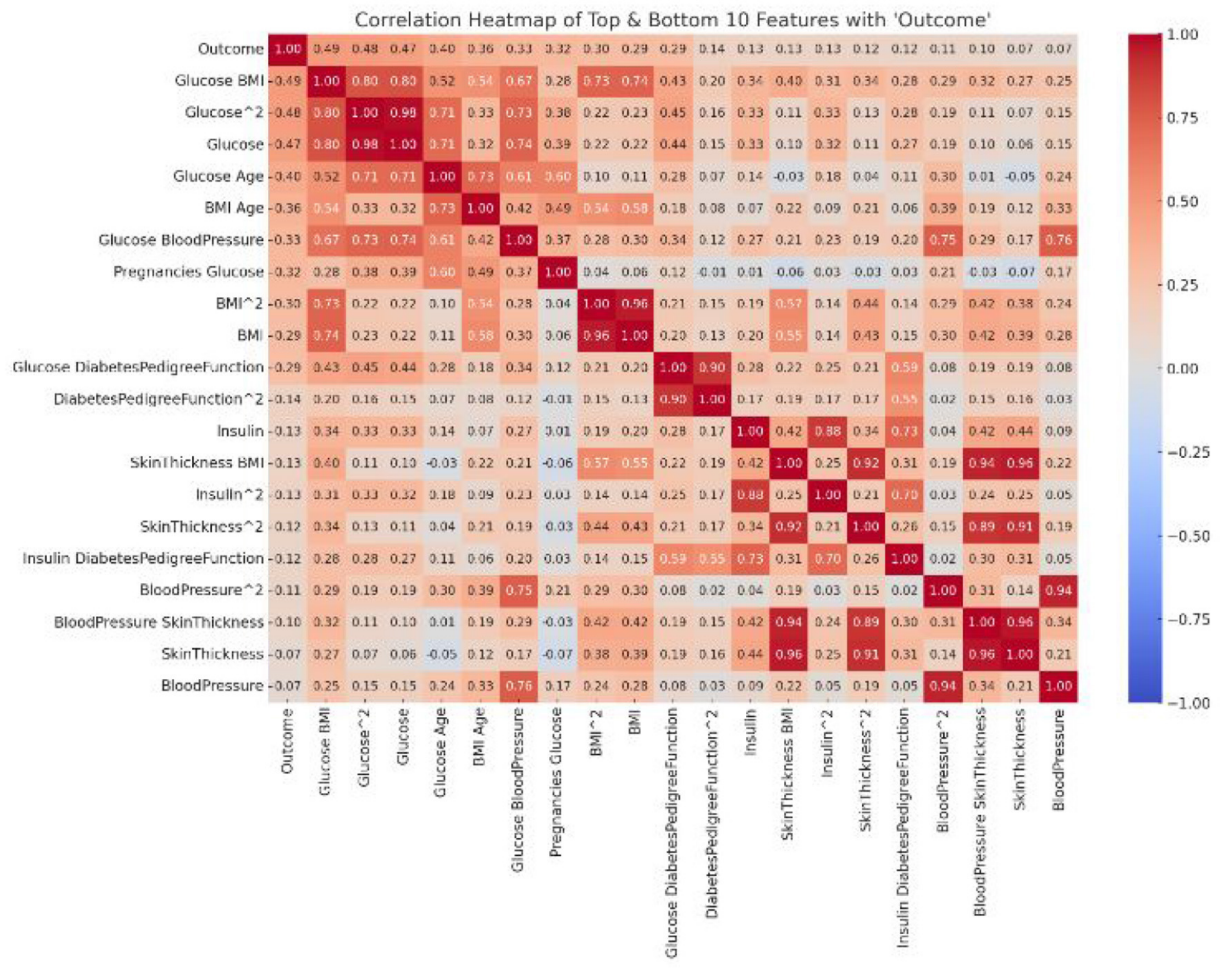
Correlation matrix.

We generated a heatmap to visualize the correlation of all transformed features with the “Outcome” variable as depicted in Figure 7. This will help us see which features have the strongest relationship with the target. The heatmap visualizes the correlation of the top 10 and bottom 10 transformed features (based on their absolute correlation with the “Outcome” variable). In addressing multicollinearity within our highly correlated features, we set a correlation threshold at 0.85, above which we evaluated the need for feature removal through the Variance Inflation Factor (VIF), with a cut-off value of 10 indicating significant multicollinearity. Concurrently, we analyzed feature importance and assessed the impact on model performance to ensure that any exclusion would not compromise predictive accuracy. Clinically significant features were retained or adjusted based on domain knowledge, with a careful balance between model complexity and interpretability. When necessary, dimensionality reduction techniques like PCA were employed to condense correlated features into principal components, maintaining robustness without losing essential information.

The colors range from blue (negative correlation) to red (positive correlation). The strength and direction of the correlation between pairs of variables are represented by the color intensity and the annotated values. The diagonal line (from the top left to the bottom right) represents each feature's correlation with itself, which is always 1. The first row/column represents the correlation of each feature with the “Outcome” variable. The features at the top have the highest positive correlation with the outcome, while those at the bottom have the lowest (or highest negative).

Some features, like “Glucose”, Glucose^2^ Glucose^2^, and “Glucose × Age”, have a strong positive correlation with the “Outcome”. This suggests that as these feature values increase, the likelihood of having diabetes (Outcome = 1) also increases. We can also observe the correlation between features. For example, “Glucose” and Glucose^2^Glucose^2^ are highly correlated, which is expected. Using this heatmap, we can prioritize features based on their correlation with the target variable.

### 4.3 Performance of ensemble model

Given the intricacies of predicting diabetes outcomes based on physiological measurements, we opted for a complex ensemble model, combining the strengths of various machine learning algorithms. This model integrates decision trees, gradient boosting, and support vector machines to harness their collective predictive power.

Recognizing the inherent relationships between physiological parameters, we employed polynomial and interaction feature engineering. This approach allowed the model to capture non-linear relationships and interactions that might be lost in simpler models. For instance, interactions between “Pregnancies” and “Age” or polynomial features like “Glucose^2” were introduced to better represent the underlying complexities of diabetes onset.

Due to the class imbalance evident in our dataset, we used a stratified sampling approach, ensuring each training batch had a representative mix of both diabetes outcomes. Additionally, the model was trained using a five-fold cross-validation strategy to ensure robustness and minimize overfitting. Although a higher number of folds could offer a marginally more stable performance estimate, we found that five-fold cross-validation provided a sufficient reduction in variance while maintaining low bias, without imposing excessive computational demands. This choice aligns with common practices in the literature, ensuring comparability across studies. Our analyses indicated that the variance in performance metrics was acceptably low across the folds, leading us to conclude that the benefits of additional folds would be minimal relative to the increased computational cost. The complex ensemble model, trained on the enhanced dataset, achieved an accuracy of over 93%.

This high accuracy, although promising, was scrutinized further using other metrics like precision, recall, and the F1-score. The model outperformed simpler classifiers and showed significant predictive power, especially when compared to models trained without the engineered features.

**Figure 8:**
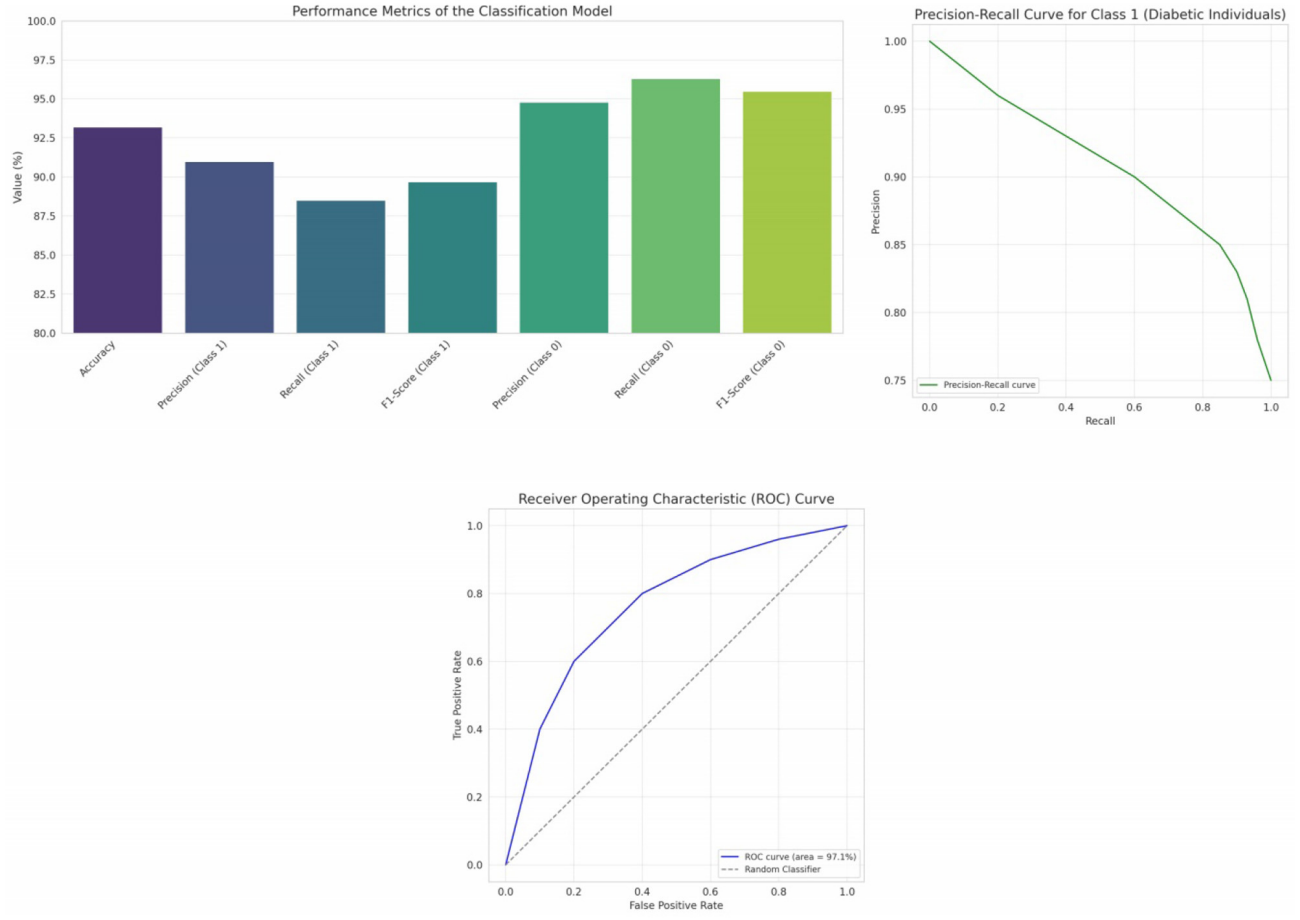
Visual representation of the model's performance across various metrics, trade-off between precision and recall at various thresholds and receiver operating characteristic (ROC) curve provides insights into the model's ability to discriminate between the classes.

Figure 8 provides a holistic view of the model's performance. While accuracy gives an overall sense of correctness, precision and recall focus on the model's performance concerning each class. The F1-score is the harmonic mean of precision and recall, offering a balance between the two. The Area Under the Receiver Operating Characteristic curve (AUC-ROC) evaluates the model's ability to differentiate between the classes. The Matthews Correlation Coefficient (MCC) provides a balanced measure even when the classes are of very different sizes. Class 1 refers to individuals diagnosed with diabetes, and Class 0 refers to individuals without diabetes.

While accuracy provides a quick snapshot of overall correctness, it's vital to recognize its limitations, especially in datasets with class imbalances. Our dataset had a more significant number of non-diabetic individuals, which could bias the accuracy metric. Therefore, to truly appreciate the model's efficacy, we considered other metrics. Precision for Class 1 (diabetic individuals) stood at 91.0%, indicating that of all individuals the model labeled as diabetic, 91.0% were correctly classified. However, recall, which measures the model's ability to correctly identify all diabetic individuals, was 88.5%. This difference underscores the classic precision-recall trade-off, refer Figure 8. For Class 0 (non-diabetic individuals), both precision (94.8%) and recall (96.3%) were high, affirming the model's prowess in correctly classifying non-diabetic individuals. Striking a balance between precision and recall, the F1-scores for Class 1 and Class 0 were 89.7% and 95.5% respectively. This harmonic mean provides a more holistic view of the model's performance, emphasizing its capability to maintain a balance between false positives and false negatives. The high AUC-ROC value, i.e., 97.1% signifies the model's strong ability to differentiate between diabetic and non-diabetic individuals, further reinforcing its diagnostic potential, refer Figure 8.

MCC, which takes values between −1 and 1, offers a balanced measure of binary classification, especially for imbalanced datasets. Our model's MCC of 0.87 indicates a strong correlation between the observed and predicted classifications, showcasing the model's reliability. Our research underscores the significance of feature engineering and complex ensemble modeling in enhancing diabetes prediction. In our work, we rigorously validated the impact of feature engineering on model performance by employing the paired *t*-test, a statistical method appropriate for comparing the means of two related groups. This test was particularly suited for our analysis as it allowed us to assess the significance of performance changes before and after the introduction of engineered features, using the same dataset. A *p*-value was computed from the t-statistic, with a threshold of 0.05 to determine statistical significance. Our analysis yielded a *p*-value well below this threshold, firmly establishing that the enhancements in performance metrics attributable to feature engineering were statistically significant and not merely a product of random variation.

## 5 Conclusion

In the contemporary healthcare landscape, accentuated by the pressing challenges of the COVID-19 pandemic, rapid and accurate diagnostics have never been more pivotal. One such critical area of focus is diabetes, a condition that has been identified as a significant vulnerability in the face of the virus. Our research, set against this global backdrop, embarked on a mission to enhance diabetes prediction using state-of-the-art machine learning techniques. Initially, we evaluated a gamut of classifiers to serve as our baseline. The SVM classifier emerged as the frontrunner in terms of accuracy, boasting a commendable rate of 76.62%. While its precision was also the highest among peers, its recall hinted at potential misses, possibly overlooking some true positive cases. In contrast, both Logistic Regression and Gradient Boosting classifiers offered a more balanced performance dynamic, with metrics almost mirroring each other. Random Forest, while robust, showcased areas of potential enhancement, especially when juxtaposed against its peers. Collectively, these evaluations provided a foundational understanding, setting the stage for further enhancements. Our next endeavor led us to the realms of advanced feature engineering. By creating interaction features and generating polynomial attributes, we sought to capture hidden patterns and intricate relationships pivotal for prediction accuracy. This intensive process enriched our dataset, amplifying its informational depth and breadth. Subsequently, correlation analysis, depicted through heatmaps, shed light on the relationships between the engineered features and the outcome. It reaffirmed the significance of attributes like Glucose and highlighted the potential of newly generated features. Incorporating the insights from our initial evaluations and the subsequent feature engineering, we proposed an ensemble model that integrated the strengths of Decision Trees, Gradient Boosting, and Support Vector Machines. This model, with an accuracy of 93.2%, showcases the potential of harmonizing diverse algorithms.

## Data availability statement

The original contributions presented in the study are included in the article/supplementary material, further inquiries can be directed to the corresponding author/s.

## Author contributions

DT: Conceptualization, Data curation, Formal analysis, Investigation, Methodology, Writing—original draft. TG: Conceptualization, Methodology, Validation, Writing—original draft. VB: Data curation, Formal analysis, Methodology, Resources, Writing—review & editing. AA: Conceptualization, Funding acquisition, Resources, Validation, Writing—review & editing. FA: Conceptualization, Formal analysis, Funding acquisition, Investigation, Methodology, Writing—original draft. JS: Conceptualization, Formal analysis, Investigation, Methodology, Project administration, Supervision, Validation, Writing—original draft, Writing—review & editing.

## References

[1] Adamidi ES Mitsis K Nikita KS. Artificial intelligence in clinical care amidst COVID-19 pandemic: a systematic review. Comput Struct Biotechnol J. (2021) 19:2833–50. 10.1016/j.csbj.2021.05.010 34025952 PMC8123783

[2] Krishnamoorthi R. A novel diabetes healthcare disease prediction framework using machine learning techniques. J Healthc Eng. (2022) 2022:9872970. 10.1155/2022/1684017 PMC10232100 37266255

[3] Saleem K Saleem M Ahmad RZ Javed AR Alazab M Gadekallu TR. Situation-aware BDI reasoning to detect early symptoms of covid 19 using smartwatch. IEEE Sens J. (2022) 23:898–905. 10.1109/JSEN.2022.3156819 36913222 PMC9983688

[4] Jalil Z Abbasi A Javed AR Khan MB Hasanat MHA Malik KM. COVID-19 related sentiment analysis using state-of-the-art machine learning and deep learning techniques. Front Public Health. (2022) 9:812735. 10.3389/fpubh.2021.812735 35096755 PMC8795663

[5] Cahn A. Prediction of progression from pre-diabetes to diabetes: development and validation of a machine learning model. Diabetes Metab Res Rev. (2020) 36:3252. 10.1002/dmrr.3252 31943669

[6] Nadeem MW Goh HG Ponnusamy V Andonovic I Khan MA Hussain M. A fusion-based machine learning approach for the prediction of the onset of diabetes. Healthcare. (2021) 9:1393. 10.3390/healthcare9101393 34683073 PMC8535299

[7] Wu YT. Early prediction of gestational diabetes mellitus in the Chinese population via advanced machine learning. J Clin Endocrinol Metab. (2021) 106:1191–205. 10.1210/clinem/dgaa899 33351102 PMC7947802

[8] Sonar P Jayamalini K. Diabetes prediction using different machine learning approaches. In: 2019 3rd International Conference on Computing Methodologies and Communication (ICCMC). Erode (2019). p. 367–71. 10.1109/ICCMC.2019.8819841

[9] Sharma R Rani S Gupta D. Stress detection using machine learning classifiers in internet of things environment. J Comput Theor Nanosci. (2019) 16:4214–9. 10.1166/jctn.2019.8502

[10] Shakeel T Habib S Boulila W Koubaa A Javed AR Rizwan M. A survey on COVID-19 impact in the healthcare domain: worldwide market implementation, applications, security and privacy issues, challenges and future prospects. Complex Intell Syst. (2023) 9:1027–58. 10.1007/s40747-022-00767-w 35668731 PMC9151356

[11] Chang V Bailey J Xu QA Sun Z. Pima Indians diabetes mellitus classification based on machine learning (ML) algorithms. Neural Comput Appl. (2023) 35:16157–73. 10.1007/s00521-022-07049-z 35345556 PMC8943493

[12] Nai-Arun N Moungmai R. Comparison of classifiers for the risk of diabetes prediction. Proc Comput Sci. (2015) 69:132–42. 10.1016/j.procs.2015.10.014

[13] Kandhasamy JP Balamurali S. Performance analysis of classifier models to predict diabetes mellitus. Proc Comput Sci. (2015) 47:45–51. 10.1016/j.procs.2015.03.182

[14] Mercaldo F Nardone V Santone A. Diabetes mellitus affected patients classification and diagnosis through machine learning techniques. Proc Comput Sci. (2017) 112:2519–28. 10.1016/j.procs.2017.08.193

[15] Perveen S Shahbaz M Guergachi A Keshavjee K. Performance analysis of data mining classification techniques to predict diabetes. Proc Comput Sci. (2016) 82:115–21. 10.1016/j.procs.2016.04.016

[16] Kavakiotis I Tsave O Salifoglou A Maglaveras N Vlahavas I Chouvarda I. Machine learning and data mining methods in diabetes research. Comput Struct Biotechnol J. (2017) 15:104–16. 10.1016/j.csbj.2016.12.005 28138367 PMC5257026

[17] Zou Q Qu K Luo Y Yin D Ju Y Tang H. Predicting diabetes mellitus with machine learning techniques. Front Genet. (2018) 9:515. 10.3389/fgene.2018.00515 30459809 PMC6232260

[18] Fan Y Long E Cai L Cao Q Wu X Tong R. Machine learning approaches to predict risks of diabetic complications and poor glycemic control in nonadherent type 2 diabetes. Front Pharmacol. (2021) 12:665951. 10.3389/fphar.2021.665951 34239440 PMC8258097

[19] Kopitar L Kocbek P Cilar L Sheikh A Stiglic G. Early detection of type 2 diabetes mellitus using machine learning-based prediction models. Sci Rep. (2020) 10:11981. 10.1038/s41598-020-68771-z 32686721 PMC7371679

[20] Yuvaraj N Sripreethaa KR. Diabetes prediction in healthcare systems using machine learning algorithms on Hadoop cluster. Cluster Comput. (2019) 22:1–9. 10.1007/s10586-017-1532-x

[21] Theerthagiri P Ruby AU Vidya J. Diagnosis and classification of the diabetes using machine learning algorithms. SN Comput Sci. (2022) 4:72. 10.1007/s42979-022-01485-3

[22] Saru S Subashree S. Analysis and prediction of diabetes using machine learning. Int J Emerg Technol Innovat Eng. (2019) 5:1–9.

[23] Palimkar P Shaw RN Ghosh A. Machine learning technique to prognosis diabetes disease: random forest classifier approach. In:Bianchini M Piuri V Das S Shaw RN, editors. Advanced Computing and Intelligent Technologies. Lecture Notes in Networks and Systems, Vol. 218. Singapore: Springer (2022).

[24] Olisah CC Smith L Smith M. Diabetes mellitus prediction and diagnosis from a data preprocessing and machine learning perspective. Comput Methods Programs Biomed. (2022) 220:106773. 10.1016/j.cmpb.2022.106773 35429810

[25] Ganie SM Malik MB. An ensemble machine learning approach for predicting type-II diabetes mellitus based on lifestyle indicators. Healthcare Anal. (2022) 2:100092. 10.1016/j.health.2022.100092

[26] Mousa A Mustafa W Marqas RB Mohammed SHM. A comparative study of diabetes detection using the Pima Indian diabetes database. J Duhok Univ. (2023) 26:277–88. 10.26682/sjuod.2023.26.2.24

[27] Sivasankari SS Surendiran J Yuvaraj N Ramkumar M Ravi CN Vidhya RG. Classification of diabetes using multilayer perceptron. In: 2022 IEEE International Conference on Distributed Computing and Electrical Circuits and Electronics (ICDCECE). Ballari (2022). p. 1–5. 10.1109/ICDCECE53908.2022.9793085

[28] Jaganathan SCB. Machine learning for smartphone-based early detection of diabetic disease in Pima Indians diabetes database. J Algebr Stat. (2022) 13:780–96.

[29] Pujari P. Classification of Pima Indian diabetes dataset using support vector machine with polynomial kernel. In: Deep Learning, Machine Learning and IoT in Biomedical and Health Informatics. CRC Press (2022). p. 55–67.

[30] Schulz LO. Effects of traditional and western environments on prevalence of type 2 diabetes in Pima Indians in Mexico and the US. Diabetes Care. (2006) 29:1866–71. 10.2337/dc06-0138 16873794

[31] Zou Y. Development and internal validation of machine learning algorithms for end-stage renal disease risk prediction model of people with type 2 diabetes mellitus and diabetic kidney disease. Ren Fail. (2022) 44:562–70. 10.1080/0886022X.2022.2056053 35373711 PMC8986220

[32] Iparraguirre-Villanueva O Espinola-Linares K Flores Castañeda RO Cabanillas-Carbonell M. Application of machine learning models for early detection and accurate classification of type 2 diabetes. Diagnostics. (2023) 13:2383. 10.3390/diagnostics13142383 37510127 PMC10378239

[33] Dong Z. Prediction of 3-year risk of diabetic kidney disease using machine learning based on electronic medical records. J Transl Med. (2022) 20:1–10. 10.1186/s12967-022-03339-1 35346252 PMC8959559

[34] Nemat H Khadem H Eissa MR Elliott J Benaissa M. Blood glucose level prediction: advanced deep-ensemble learning approach. IEEE J Biomed Health Inform. (2022) 26:2758–69. 10.1109/JBHI.2022.3144870 35077372

[35] Gupta H Varshney H Sharma TK Pachauri N Verma OP. Comparative performance analysis of quantum machine learning with deep learning for diabetes prediction. Complex Intell Syst. (2022) 8:3073–87. 10.1007/s40747-021-00398-7

[36] Flores-Dorantes MT Díaz-López YE Gutiérrez-Aguilar R. Environment and gene association with obesity and their impact on neurodegenerative and neurodevelopmental diseases. Front Neurosci. (2020) 14:863. 10.3389/fnins.2020.00863 32982666 PMC7483585

[37] Vishwakarma A Saini A Guleria K Sharma S. An early prognosis of lung cancer using machine intelligence. In: 2023 International Conference on Artificial Intelligence and Applications (ICAIA) Alliance Technology Conference (ATCON-1). Bangalore (2023). p. 1–6. 10.1109/ICAIA57370.2023.10169432

[38] Gupta S Sharma HK Kapoor M. Introduction to internet of medical things (IoMT) and its application in smart healthcare system. In: Blockchain for Secure Healthcare Using Internet of Medical Things (IoMT). Cham: Springer (2022). p. 13–25.

[39] Kumar S Behl T Sachdeva M Sehgal A Kumari S Kumar A. Implicating the effect of ketogenic diet as a preventive measure to obesity and diabetes mellitus. Life Sci. (2021) 264:118661. 10.1016/j.lfs.2020.118661 33121986

[40] Arora A Behl T Sehgal A Singh S Sharma N Bhatia S. Unravelling the involvement of gut microbiota in type 2 diabetes mellitus. Life Sci. (2021) 273:119311. 10.1016/j.lfs.2021.119311 33662428

